# Evidence-Based African First Aid Guidelines and Training Materials

**DOI:** 10.1371/journal.pmed.1001059

**Published:** 2011-07-19

**Authors:** Stijn Van de Velde, Emmy De Buck, Philippe Vandekerckhove, Jimmy Volmink

**Affiliations:** 1 Centre of Expertise, Belgian Red Cross-Flanders, Mechelen, Belgium; 2 Faculty of Health Sciences, Stellenbosch University, Cape Town, South Africa

## Abstract

Stijn Van de Velde and colleagues describe the African First Aid Materials project, which developed evidence-based guidelines on administering first aid in the African context as well as training materials to support the implementation of the guidelines.

**Summary Points**

- In sub-Saharan Africa, much disease and injury can be addressed by emergency care. First aid training has been promoted as an inexpensive way to save lives. However, first aid training in sub-Saharan Africa is often based on handbooks prepared outside of the continent that are not adapted to the African context.
- We formulated evidence-based guidelines that comprehensively address how basic first responders should be trained to manage emergency situations in an African context. The guidelines focussed on first aid interventions requiring minimal or no equipment.
- Two assessors reviewed the evidence, giving special attention to studies on African cultural remedies and preferences. A multidisciplinary panel of 11 African experts discussed each recommendation until they reached agreement. GRADE was used to grade the quality of evidence and strength of recommendations. Peer reviewers gave feedback on the guidelines.
- These new guidelines formed the basis of a flexible didactic materials kit that is available to those involved in first aid training programmes in Africa. An implementation guide is also available to help tailor the training materials to the local context and target group.

This Health in Action report describes the African First Aid Materials project (AFAM, http://www.afam.redcross.be/). This project developed evidence-based guidelines on how basic first responders should be trained to manage emergency situations in an African context. The project also includes the development of training materials to support the implementation. The objective of this manuscript is to inform educators and health care professionals about these guidelines and training materials and to promote their use when developing first aid training programmes for sub-Saharan Africa.

## The Burden of Emergencies

In sub-Saharan Africa, 41% of all deaths and 39% of the morbidity burden can potentially be addressed by emergency care [1],[2]. Road traffic injuries may dramatically increase this burden in the coming years [3]. In many emergencies, prompt and adequate care can increase the likelihood of survival and recovery. Pre-hospital care is a vital initial step; however, it is often unavailable in sub-Saharan Africa. When an emergency occurs, basic first responders often constitute the sole source of initial help, and emergency transport often consists of a ride in taxis, private cars, or public transport [4],[5]. Studies in Ghana and Uganda demonstrated that training basic first responders leads to improved provision of help in the absence or anticipation of medical care [6]–[8]. Therefore, the World Bank has promoted first aid training as an inexpensive way to save lives in sub-Saharan Africa.

The distinct African burden of disease and injury, the limited access to formal health care, the strong embeddedness of cultural remedies, and poverty all call for specific first aid guidelines. However, first aid training in sub-Saharan Africa is often based on handbooks prepared outside of the continent that are not adapted to the African context. Although well-intentioned, such resources sometimes lead to misdirected, inadequate, or even harmful training instructions. Several African Red Cross National Societies expressed the need for first aid materials adapted to the African context. We are not aware of any evidence-based reference that comprehensively addresses how basic first responders should be trained to manage emergency situations in an African context. Since evidence-based reviews with relevance to low- and middle-income countries are scarce, this added to the importance of our work [9]. Our guidelines are intended to guide those responsible for first aid programmes and to form the basis of African training materials. We defined first aid as “appropriate and beneficial help to a suddenly ill or injured person which is initiated as soon as possible and continued until that person has recovered or medical care is available”. These guidelines focussed on first aid interventions requiring minimal or no equipment.

## Summary of Methods to Develop the Guidelines

The guidelines represent the consensus judgment of an expert panel taking into account findings from systematic reviews on the following topics: stroke, heart attack, fever, fits, diarrhoea, rash, wounds and injuries, poisoning, and emergency childbirth. This topic selection was based on published injury and disease statistics for sub-Saharan Africa [1],[2]. For the topics of choking, unconsciousness, and heart arrest, we used the evidence-based guidelines with instructions for basic first responders published by the International Liaison Committee on Resuscitation (ILCOR) [10].

### Search Strategy to Identify Relevant Evidence

Between February and April 2009, two assessors (SVDV, ED) identified studies assessing the effects and feasibility of first aid procedures for the selected emergency situations. To identify evidence-based guidelines, they searched the Guidelines International Network (GIN) database, the WHO Library Database, and African Index Medicus using the search terms detailed in the technical appendix (Text S1). MEDLINE, EMBASE, *The Cochrane Library*, BestBets, and African Index Medicus were searched to identify existing systematic reviews. When necessary, they updated the systematic reviews or started a new systematic review of intervention studies. In the case where no intervention studies were found, they also searched for prospective observational studies. A separate review was conducted for cross-sectional surveys and qualitative research on African cultural remedies and preferences in relation to the included emergency situations (criteria for considering studies for inclusion are detailed in Text S1).

### Selection of Studies

Based on pre-defined selection criteria (detailed in Text S1), two authors selected studies from the titles and abstracts of all the retrieved references. They then screened full texts and further excluded irrelevant studies. Reference lists of selected studies were checked to identify further studies.

### Quality Assessment, Data Extraction, and Evidence Synthesis

The authors assessed the quality of evidence according to Grading of Recommendations Assessment, Development and Evaluation (GRADE), which takes into account limitations in design, inconsistency, indirectness, imprecision, and likelihood of publication bias. The quality of evidence could either be high, moderate, low, or very low [11]. Data on methodology, participants, intervention, comparison, and outcomes were extracted into evidence profiles. A descriptive summary of the evidence and draft recommendations were then prepared for consideration by the expert panel.

### Expert Panel

The expert panel included four African specialists with expertise in evidence-based medicine and primary care or emergency medicine, five representatives of African Red Cross societies (managers and first aid trainers), and an expert in medical anthropology. The co-director of the South-African Cochrane Centre (JV), part of the international Cochrane Collaboration, served as the chairperson.

The expert panel met for the first time in September 2009. The aims of this meeting were to identify any potential conflict of interest, to explain the guidelines development methods, and to present the draft evidence synthesis and recommendations. The recommendations were accompanied with photos and drawings that illustrated the conditions and actions. In the next few weeks, the experts revised the draft documents and sent their feedback by email. A checklist based on items from the AGREE [12], GLIA [13], and DISCERN [14] instruments guided the experts through this revision.

A follow-up two-day consensus meeting was held in October 2009. This meeting aimed to reformulate the recommendations until agreement was reached among the full expert panel and to decide on the strength of each recommendation (strong or weak) according to GRADE [11]. The panel discussed each recommendation, taking into account the evidence, the feedback from the preparatory revision, practical experience, and contextual factors. The strength of the recommendation was influenced by the benefits and harms, quality of evidence, applicability, and preferences of the population. If no relevant research evidence was found, the panel based the recommendations on what was considered good practice. In that case, no grade of recommendation was given. The meeting was audio recorded to capture all the comments and decisions in the meeting minutes.

### Peer Review

In February 2010, four reviewers who specialised in emergency medicine, traumatology, paediatrics, and obstetrics were invited to give feedback on the guidelines and on the draft of the didactical material. The chair of the guidelines development panel then considered the responses and improvements were made.

### Internal Validation

The final version of the guidelines was approved electronically by the expert panel.

## Evidence and Recommendations

Overall, we screened 24,000 references and selected 143 publications to provide the evidence base for the guidelines. The systematic reviews allowed us to learn about practices that try to make the best use of the limited resources available. Examples of such practices that were considered by the panel include ash for hand washing [15],[16], homemade oral rehydration solutions for diarrhoea [17], early breastfeeding for emergency childbirth [18],[19], fever measurement by touch [20], tepid sponging for fever [21], boiled and cooled water for wound cleansing [22], application of honey or aloe vera, and dressings from banana leaf or potato peel for burns [23]–[27].

The complete guidelines (Text S2) include a narrative synthesis of the results of the systematic reviews and recommendations for each included topic. We have chosen to report here the evidence and recommendations for snakebites as a sample of the guidelines.

### First Aid for Snakebites

In sub-Saharan Africa, snakebites are a neglected health problem with an important burden of disease for which many victims never receive formal medical care [28],[29]. The application of an elastic bandage combined with immobilisation is an often recommended first aid technique for snake bites. The bandage should be applied tightly, but applying it not tight enough or too tight is ineffective and sometimes harmful [30]. Two studies indicate that applying a compression bandage over a firm cloth pad is effective; however, this technique increases the risk of creating an arterial tourniquet [31],[32]. Two studies indicate that motivated persons have difficulty applying a compression bandage at the correct pressure, even after intense training [33],[34]. One study indicates that walking increases the transit of venom [30]. One study indicates that immobilisation can be taught to basic first responders [34]; however, a field study indicates that after receiving the instruction to immobilise bitten limbs, this was only done properly in a minority of cases [35].

Based on these findings, the panel recommended immobilisation of the bitten limb without application of an elastic bandage. The level of evidence is low to very low with a weak grade of recommendation. Additional recommendations are to stop the bitten person from moving, calming the person, taking off any rings, watches, or tight clothing that may cut off blood flow because of swelling, and taking actions to obtain medical help.

## Moving from Guidelines to Training Materials

Writing guidelines is only the beginning and should be followed by measures to support implementation in practice. Therefore, these new guidelines formed the basis of a flexible training materials kit that comes on a DVD. This DVD will be available to those responsible for first aid training programmes in Africa. The training materials include an electronic version of the manual illustrated with hundreds of quality illustrations including youths, adults, and elderly people from multiple ethnic and religious backgrounds. The manual is available in English, French, and Portuguese and describes for every condition the most important signs to look for and actions to take. Currently, didactical posters and videos are being developed to demonstrate the different procedures. Providers of first aid courses can pick what they need from the DVD to compose their first aid courses for their specific target audience.

Although the guidelines attempted to be generic for sub-Saharan Africa, there are local specificities that need to be addressed in order to apply the training materials. For example, misunderstandings and mistrust regarding health information may arise when the biomedical approach is at odds with local perceptions and management. Advice that incorporates local terms, perceptions, and preferences is considered to be more convincing [36]. Therefore, an implementation guide is also available to assist those responsible for first aid programmes on ways to adapt the training materials.

Between June and December 2010 we piloted the training materials and implementation guide in Uganda and Swaziland. These pilots include focus group discussions on whether the illustrations are understood to evaluate clarity, and field-testing the feasibility of more complex instructions. For this purpose, individuals with different levels of experience in first aid (from novice to trainer) were asked to perform specific first aid tasks under different test conditions. The test conditions included performing the task when being provided with drawings only, with drawings and written instructions only, or after the demonstration by a trainer. An observer recorded if the participants completed the task correctly or incorrectly.

## Where Is the Project Heading Next?

Implementation of AFAM in the Red Cross national societies from Namibia, Mozambique, South Africa, Malawi, Uganda, Swaziland, the Comoros, and Kenya is planned. We are also developing a strategy to formulate a similar set of guidelines and training materials for South Asia. Furthermore, because first aid training is often used as an entry point for primary prevention, we have commenced a project to formulate evidence-based preventive measures that can be used in addition to the first aid guidelines described in AFAM.

## Supporting Information

Text S1 Further details of methods.
(DOCX)

Text S2 African First Aid Materials: Guidelines (Belgian Red Cross-Flanders).
(PDF)

## Abbreviations

AFAM: African First Aid Materials
GRADE: Grading of Recommendations Assessment, Development and Evaluation
