# Supplementary material for: Evidence-Based African First Aid Guidelines and Training Materials
Source: PLoS Med. 2011 Jul 19;8(7):e1001059. doi: 10.1371/journal.pmed.1001059 (PMC3139663; doi:10.1371/journal.pmed.1001059)
Supplement: Text S1 — Further details of methods. (DOCX) [file pmed.1001059.s001.docx]

**Text S1**

This file contains further details of methods to accompany the paper Evidence based African first aid guidelines and training materials by Stijn Van de Velde, Emmy De Buck, Philippe Vandekerckhove, and Jimmy Volmink.

**Core search terms used to identify relevant studies**

First Aid; Community Health Aides; Emergency Treatment; Emergency Medical Services; Emergency Service, Hospital; Poison Control Centres; Transportation of Patients; Primary Health Care; Acute disease; Emergencies; Wound healing; Medicine, African Traditional; Infection Control; Stress Disorders, Traumatic, Acute; Dehydration; Diarrhoea; Pneumonia; Dyspnoea; Malaria; Fever; Seizures; Epilepsy; Seizures; Alcohol Withdrawal Delirium; Measles; Stroke; Myocardial Infarction; Chest Pain; Haemorrhage; Lacerations; Wounds, Non penetrating; Soft Tissue Injuries; Sprains and Strains; Tendon Injuries; Contusions; Dislocations; Fractures, Bone; Spinal Cord Injuries; Spinal Injuries; Neck Injuries; Back Injuries; Craniocerebral Trauma; Arm Injuries; Athletic Injuries; Hand Injuries; Hip Injuries; Leg Injuries; Thoracic Injuries; Wounds, Penetrating; Foreign Bodies; Amputation, Traumatic; Shock, Traumatic; Burns; Bites and Stings; Poisoning; Labour, Obstetric; Parturition; Bandages; Irrigation

**Selection criteria for the review questions about effectiveness and feasibility of first aid procedures**

• Population: Sick or injured persons or healthy volunteers.

• Intervention: Studies on help provided by basic first responders, lay caregivers, community health workers, or healthcare professionals, where the interventions are feasible for extrapolation to basic first responders, and studies on diagnostic procedures based on clinical signs/symptoms.

We excluded interventions that require special equipment or competences or interventions that do not take place during the acute phase and which can be considered as aftercare.

• Outcome: Health outcome measures, diagnostic values, measures of performance by basic first

responders or lay caregivers, adverse effects.

• Design: For a guideline or review to be included we used the following criteria: the inclusion/exclusion criteria are reported; the search was adequate; the included studies are synthesised; the validity of the included studies was assessed; sufficient details about the individual included studies are presented. These criteria are based on the DARE criteria (www.york.ac.uk)

• Language: English, French, Dutch, Afrikaans or German.

**Selection criteria for the review question about cultural remedies or preferences of Africans:**

• Population: Studies done in Sub Saharan Africa with basic first responders, lay caregivers,

or community health workers.

• Intervention: No criteria.

• Outcomes: Perceived causes/mechanisms of treatment, treatment seeking behaviour, home

treatment, traditional treatment, health outcome, adverse effects, effects of health education.

• Time: Studies not older than 5 years.

•Design: We included cross-sectional surveys and qualitative research and excluded Epidemiological

studies on incidence/prevalence of injury or illness.

• Language: English, French, Dutch, Afrikaans, or German.
