# Supplementary material for: Evidence-Based African First Aid Guidelines and Training Materials
Source: PLoS Med. 2011 Jul 19;8(7):e1001059. doi: 10.1371/journal.pmed.1001059 (PMC3139663; doi:10.1371/journal.pmed.1001059)
Supplement: Text S2 — African First Aid Materials: Guidelines (Belgian Red Cross-Flanders). (PDF) [file pmed.1001059.s002.pdf]

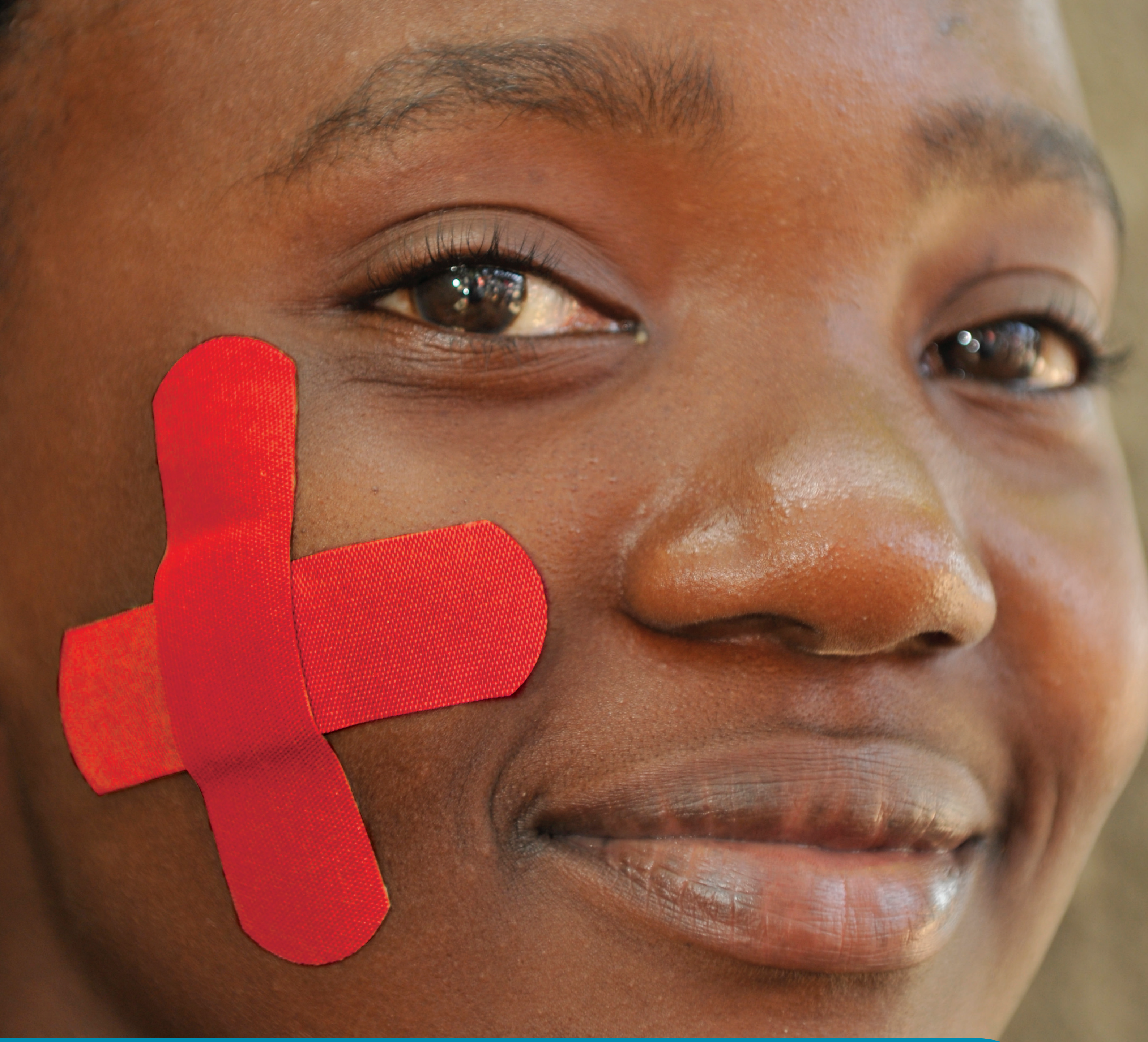

African first aid materials

EVIDENCE BASED

Belgian Red Cross
Flanders

Guidelines

Table of contents

Introduction ..... p. 2

Methodology ..... p. 3

Organisation of the guideline ..... p. 6

Basic principles for management of an emergency ..... p. 7

- *Dealing with an emergency ..... p. 7*
- *Stress in an emergency ..... p. 10*
- *Protecting from infection ..... p. 10*
- *Basic needs ..... p. 11*

Sudden illness ..... p. 14

- *Facial weakness, arm weakness and speech problems ..... p. 14*
- *Chest discomfort ..... p. 16*
- *Choking ..... p. 17*
- *Fainting or unconsciousness ..... p. 19*
- *No breathing ..... p. 21*
- *Fever ..... p. 23*
- *Fits ..... p. 26*
- *Diarrhoea ..... p. 27*
- *Rash ..... p. 29*

Injuries ..... p. 30

- *Severe bleeding ..... p. 30*
- *Wounds with bullets or objects in a cut ..... p. 32*
- *Snake, scorpion or spider bite or sting ..... p. 33*
- *Burns ..... p. 35*
- *Injury to head, neck, or back ..... p. 37*
- *Broken or dislocated limbs ..... p. 39*
- *Injury to muscles or joints ..... p. 41*
- *Eye injury ..... p. 42*
- *Bite wounds ..... p. 44*
- *Nose bleed ..... p. 45*
- *Cuts and grazes ..... p. 46*
- *Bee or wasp stings ..... p. 48*

Poisoning ..... p. 49

Emergency childbirth ..... p. 50

Acknowledgements and references ..... p. 54

Introduction

Every year thousands of people are trained in first aid around the world. The Red Cross is the main provider of this training, and first aid lies at the heart of the Red Cross movement.

In 2005, Belgian Red Cross-Flanders spearheaded an initiative (EFAM, European First Aid Manual), together with a group of European experts, to update training for basic first responders according to the latest medical and scientific data. This was done using evidence-based methodology to identify effective interventions and interventions that were dated, ineffective or even harmful. This led to the publication of validated European first aid guidelines[1] and the European First Aid Manual (www.efam.be). So far, more than thirty organisations have a licence to use EFAM as the basis for their first aid courses, meaning in effect that EFAM is also harmonising first aid training across Europe.

Several African Red Cross National Societies expressed the need for first aid materials adapted to the African context. In addition the World Bank advocates training basic first responders in how to respond to medical emergencies as a low-cost opportunity that can decrease the burden of disease and injury in Sub-Saharan Africa[2]. Such training can lead to reduced delays in recognising danger signs and seeking medical help, and to improved provision of help in the absence or anticipation of medical care[3;4]. The distinct African burden of disease and injury, the limited access to formal healthcare, the strong embeddedness of cultural remedies, and poverty all call for specific first aid guidelines.

To date, we know of no single evidence-based reference that comprehensively addresses how basic first responders should be trained to manage emergency situations in an African context. This led us to a project to use the EFAM experience to help develop evidence-based first aid guidelines and materials specifically directed at the African context.

The purpose of this project is to help decrease the burden of disease and injuries for Sub-Saharan Africa countries. Our objective is to develop guidelines on the content of first aid training programmes for basic first responders in Sub-Saharan Africa. The guidelines are intended to provide guidance and support to those responsible for first aid programmes. We define first aid as ‘appropriate and beneficial help to a suddenly ill or injured person which is initiated as soon as possible and continued until that person has recovered or medical care is available’. This guideline assumes first aid requiring minimal or no equipment.

Since evidence-based reviews with relevance for low and middle income countries are very limited,[5] this opens up possibilities to learn about successful practices that make the best of limited resources.[6] These new guidelines will be the basis of a flexible didactic materials kit that will be available to those responsible for first aid training programmes in Africa. The didactic materials kit comes on a DVD which provides texts and hundreds of quality African illustrations including youths, adults and elderly people from multiple ethnic and religious backgrounds. The text is available in English, French and Portuguese. An implementation guide is available to assist those responsible for first aid programmes.

Methodology

Methods to develop guidelines need to be rigorous and transparent. This is especially important so that the guidelines are not subject to potential biases of guideline development and that users have confidence in its validity. However, formal guideline development methods often require a lot of time and resources and two-year projects are common.[7] Practical concerns about the sustainability of such time- and resource-consuming guideline development methods have been raised[8] and made us use a more pragmatic development process.

Guideline expert panel

We composed a multidisciplinary expert panel of 10 African experts, including five specialists and five representatives of African Red Cross societies. Specialists had expertise in evidence-based medicine and primary care or emergency medicine and came from countries in Sub-Saharan Africa. Representatives of African Red Cross societies included managers and first aid trainers. An expert in medical anthropology also participated in the panel. Two experienced reviewers participated to develop the evidence profiles. The co-director of the South-African Cochrane Centre chaired the panel.

Formulating key questions

Based on published injury and disease statistics for Sub-Saharan Africa[9;10], we selected the most critical topics with relevance for first aid. The reviewers proposed a list of topics to the chair, who decided on the final scope:

- **Injuries:** severe bleeding; nose bleed; cuts and grazes; human or animal bite wounds; bee or wasp stings; spider, scorpion or snake bites; penetrating wounds; burns; eye wounds; broken bones; injuries to muscles and tendons; head injuries; spinal injuries; poisoning.
- **Communicable conditions:** fever; malaria; pneumonia; measles; diarrhoea.
- **Non-communicable conditions:** choking; unconsciousness; cardiac arrest; heart infarction; stroke; epilepsy; alcohol withdrawal delirium.
- **Maternal conditions:** emergency childbirth.

In relation to the emergency situations included, we subsequently formulated key questions to be addressed. A first key question dealt with the effectiveness and feasibility of first aid procedures for the selected emergency situations. We subdivided this question for each emergency into a) initial assessment, b) management, c) criteria for seeking medical help. A second question dealt with African cultural remedies and preferences in relation to the included emergency situations.

Systematic review

We used a stepwise approach to identify the evidence following the hierarchy of study designs. This means that we searched for guidelines, systematic reviews, intervention studies and prospective observational studies in descending order. If we found an eligible study from a higher evidence level, the following step focused only on studies from a lower evidence level published thereafter. We only searched for prospective observational studies in case no other evidence was found. We searched for:

- Guidelines in the Guidelines International Network database, the WHO Library Database, African Index Medicus[11], Medline, and Embase;
- Systematic reviews in Medline, Embase, Cochrane Library, DARE, BestBets, and African Index Medicus;
- Intervention studies in Medline, Embase, Central, and African Index Medicus;
- Prospective observational studies in Medline, Embase, and African Index Medicus.

We searched these databases between February and April 2009.

To ensure that we did not miss studies with relevance to Africa by using methodological filters or time restrictions, we searched Pubmed and Embase using only a geographical filter for Sub-Saharan Africa[12]. For studies on African cultural remedies and preferences we searched in Medline, Embase, and African Index Medicus for studies published within the last five years. We used the following core search terms:

> First Aid; Community Health Aides; Emergency Treatment; Emergency Medical Services; Emergency Service, Hospital; Poison Control Centres; Transportation of Patients; Primary Health Care; Acute disease; Emergencies; Wound healing; Medicine, African Traditional; Infection Control; Stress Disorders, Traumatic, Acute; Dehydration; Diarrhoea; Pneumonia; Dyspnoea; Malaria; Fever; Seizures; Epilepsy; Seizures; Alcohol Withdrawal Delirium; Measles; Stroke; Myocardial Infarction; Chest Pain; Haemorrhage; Lacerations; Wounds, Non penetrating; Soft Tissue Injuries; Sprains and Strains; Tendon Injuries; Contusions; Dislocations; Fractures, Bone; Spinal Cord Injuries; Spinal Injuries; Neck Injuries; Back Injuries; Craniocerebral Trauma; Arm Injuries; Athletic Injuries; Hand Injuries; Hip Injuries; Leg Injuries; Thoracic Injuries; Wounds, Penetrating; Foreign Bodies; Amputation, Traumatic; Shock, Traumatic; Burns; Bites and Stings; Poisoning; Labour, Obstetric; Parturition; Bandages; Irrigation

After defining the inclusion and exclusion criteria, we selected studies from the titles and abstracts of all the retrieved references. We then screened full texts and further excluded irrelevant studies.

For the review question about effectiveness and feasibility of first aid procedures, we used the following criteria

- **Population:** Sick or injured persons or healthy volunteers.
- **Intervention:** We included studies on help provided by basic first responders, lay caregivers, community health workers, or healthcare professionals, if the interventions are feasible for extrapolation to basic first responders, and included studies on diagnostic procedures based on clinical signs/symptoms. We excluded interventions that require special equipment or competences or interventions that do not take place during the acute phase and which can be considered as aftercare.
- **Outcome:** Health outcome measures, diagnostic values, measures of performance by basic first responders or lay caregivers, adverse effects.
- **Design:** For a guideline or review to be included we used the following criteria: the inclusion/exclusion criteria are reported; the search was adequate; the included studies are synthesised; the validity of the included studies was assessed; sufficient details about the individual included studies are presented. These criteria are based on the DARE criteria (www.york.ac.uk)
- **Language:** English, French, Dutch, Afrikaans or German.

For the review question about cultural remedies or preferences of Africans we used the following criteria:

- **Population:** Studies done in Sub Saharan Africa with basic first responders, lay caregivers, or community health workers.
- Intervention: No criteria.
- **Outcomes:** Perceived causes/mechanisms of treatment, treatment seeking behaviour, home treatment, traditional treatment, health outcome, adverse effects, effects of health education.
- Time: studies not older than 5 years.
  Design: We included cross-sectional surveys and qualitative research and excluded Epidemiological studies on incidence/prevalence of injury or illness.
- **Language:** English, French, Dutch, Afrikaans, or German.

By checking the reference lists of selected studies we were able to include studies that were not retrieved in the initial search. A limitation of this guideline is that we did not search for studies written in Portuguese or studies published in non-indexed African Journals.

We used the quality criteria of the Cochrane Effective Practice and Organisation of Care Review Group[13] for intervention studies, the quality criteria of the Dutch Cochrane Centre for cohort studies[14], and Quadas for diagnostic studies[15].

Overall we screened 24,000 references and selected 143 publications in the guideline.
We did not perform a systematic review for cardiopulmonary resuscitation and choking, because recent evidence-based guidelines with instructions for first responders are available[16].

Data synthesis

We extracted data on methodology, participants, intervention, comparison, and outcomes and tabulated evidence profiles. Draft recommendations and didactic material were prepared before the meeting of the guideline development group. We graded the quality of evidence and strength of recommendations in accordance with the GRADE system[17]. The GRADEprofiler system facilitated the determination of the quality of evidence.

GRADE divides the quality of evidence into either:

- **High** = further research is very unlikely to change our confidence in the estimate of effect;
- **moderate** = further research is likely to have a significant impact on our confidence in the estimate of effect and may change the estimate;
- **low** = further research is very likely to have a significant impact on our confidence in the estimate of effect and is likely to change the estimate;
- **very low** = any estimate of effect is very uncertain.

Within the guideline, the letters A, B, C, or D summarise high, moderate, low or very low quality of evidence respectively.

The strength of the recommendation is influenced by the benefits and harms, quality of evidence, applicability, and preferences of the population[18].

- A **strong** recommendation means 'do it', as most people in that situation would want or should receive the recommended action.
- A **weak** recommendation means 'probably do it', as there is some uncertainty regarding the most appropriate action and different choices may be appropriate.
- If no relevant research evidence was found, the panel based the recommendations on what is considered good practice. In that case no grade of recommendation is given.

Panel meeting

We organised a one-day introductory panel meeting to present the draft recommendations and didactical material, and to clarify the evidence-based methods and consensus procedures. During a two-day consensus meeting, the panel discussed each recommendation until they reached agreement.

Consultations

We invited peer reviewers including medical specialists in emergency medicine, traumatology, paediatrics, and gynaecology to give feedback on each guideline statement and on the draft of the didactical material. The chair of the guideline development panel then considered the responses.

Pilot test

We organised a pilot test of the training materials in Uganda and Swaziland to get the perspectives of Africans on AFAM. This pilot included focus group discussions on whether the illustrations were understood when presented without further explanation to evaluate clarity, and field-testing the skills learned after training with AFAM to evaluate the feasibility of more complex guidelines.

Internal validation

The final version of the guidelines and the didactical material was circulated electronically and approved by the panel members.

Guideline update

These guidelines will be updated by 2015.

Organisation of the guideline

The AFAM Guidelines are intended as a tool to dessiminate the guidelines and the evidence base behind it. We stress that these Guidelines are not a first aid manual and it is assumed that the reader knows the basics of first aid. Illustrations and details on how to perform specific techniques are not included in these Guidelines. For a full description and illustrations of the situations and techniques the reader should consult the full African First Aid Materials. AFAM describes the most important actions and illustrated them with drawings. The illustrations in AFAM are gender and age balanced and reflect ethnic and religious diversity.

AFAM is divided into five main sections:

- Basic principles for management of an emergency
- Sudden illness
- Injuries
- Poisoning
- Emergency childbirth

To make it practical for basic first responders we classified the conditions according to the most important signs instead of basing them on a diagnosis.

With the purpose of writing gender sensitive , we alternate in the Guidelines between using “he” or “she”.

Every chapter contains several boxes that summarise key information:

- The box 'When to seek medical help' lists criteria for seeking medical attention in a way that minimises the risk of disregarding emergency cases.
- The box 'Caution' highlights instructions to avoid further harm.

Each condition is linked to the evidence that forms the basis for the recommendations. In addition, we provide information on African cultural remedies and preferences linked to the condition.

AFAM is a generic instrument. Before implementing the guideline or materials in a target training group, we advise a field-test to identify local beliefs, customs, terms, expressions and the baseline first aid knowledge. This enables it to be considerate and tailored to the local context and baseline first aid knowledge. An implementation guide is available to assist those responsible for first aid programmes.

Additional information

- Health education programmes in Africa must address important cultural issues in order to be effective. The biomedical approach sometimes deviates from local perceptions and management, which can lead to mistrust[19]. Misunderstandings may arise when biomedical terms used in health information differ from local terms[20]. Advice that incorporates local terms, perceptions and preferences is considered to be more convincing[19].

- Treatment is often sought in line with a perceived cause for the disease and mechanism of treatment[19;21-24]. A sudden or severe illness is regularly linked to a supernatural cause[25]. Changing perceptions of biomedical causes of disease might change the treatment seeking behaviour[21]. The fear of some types of medical treatment can also be a reason for not seeking medical help[21;24].

- Educational programmes often focus on mothers as they tend to be the primary caregivers at the household level. However, in the household men often take the decisions or hold the financial resources. Therefore, health education should target men as well as women[26].

Basic principles for management of an emergency

Dealing with an emergency

Emergency situations vary greatly but there are four main steps that always apply:

- *Step 1 Make the area safe.*
- *Step 2 Evaluate the injured person's condition.*
- *Step 3 Seek help.*
- *Step 4 Give first aid.*

Step 1 Make the area safe

Your *own safety* should always come first. As a first aider, you should:

- Try to find out what has just happened.
- Check for any danger: is there a threat from traffic, fire, electricity cables, etc.?
- Never approach the scene of an accident if you are putting yourself in danger.
- Do your best to protect both the injured person(s) and other people on the scene.
- Be aware that the property of the injured person is at risk. Theft can occur, so mind your safety.
- Seek police or emergency help if an accident scene is unsafe and you cannot offer help without danger to yourself.

In case of *road accidents*, as a first aider, you should:

- Always follow the traffic rules.
- Ask other people to warn traffic.
- Consider seeking help from the police or emergency services.
- Do not allow anybody to smoke near an accident.
- Switch off the engine of every car involved in the accident.
- Try to apply the handbrake of cars involved in the accident to prevent them from moving. You can also put something against the tyres to prevent rolling.
- Place a warning triangle at a good distance, at least 30 meters to either side of the accident, to warn traffic.
- If a warning triangle is not available, use a warning sign that is approved or permitted by the law of the country.
- Do not forget to clear the warning signs afterwards.

As a general rule, the injured person should *not be moved* from the scene of an accident. Any movement may make the injury worse if there has been a head, neck, back, leg or arm injury.

Only move an injured person if:

- The injured person is in more danger if he is left there.
- The situation cannot be made safe.
- Medical help will not arrive soon.
- You can do so without putting yourself in danger.

How to move an injured person?

There are different techniques possible for moving injured persons. Which technique is most appropriate depends on the situation.

- Use tools that you have at hand to free an entrapped person.
  ! Be careful not to cause harm to the trapped person.
- If the injured person is conscious, explain what you are going to do.
  Ask the injured person to follow your instructions.
- Try not to twist the head, neck or body. If possible, support the injured person's neck.
  If someone has an injured spine, movement may cause further damage.
- It’s important to move the injured person quickly but try to keep the injured person’s body as still as possible.
- Move the injured person to the nearest safe place or to a place where he can get help.

For a full description of the technique "moving an ill or injured person" the reader should consult the full African First Aid Materials.

Step 2 *Evaluate the condition of the ill or injured person*

If the area is safe, you can evaluate the ill or injured person’s condition. Always check that he is conscious and breathing normally. Situations in which consciousness or breathing are impaired are often life threatening. Below we list other important situations that are life threatening or could become so if left untreated.

Related to sudden illness:
Sudden facial weakness, arm weakness or speech problems (see p. 14)
Chest discomfort (see p. 16)
Not being able to stand or sit up (see chapter 'Fever' p. 23)
Great sleepiness, confusion (see chapters 'Fever' p. 23, 'Diarrhoea' p. 27 and 'Rash' p. 29)
Not being able to drink, vomiting everything, diarrhoea, dehydration (see chapters 'Fever' p. 23, 'Diarrhoea' p. 27 and 'Rash' p. 29)
Spontaneous bleeding (see chapters and 'Fever' p. 23 and 'Rash' p. 29)
Fits (see p. 26)

Related to injuries:
Severe bleeding (see p. 30)
Wounds with bullets or objects in a cut (see p. 32)
Severe burns (see p. 35)
Snake bite (see p. 33)
Injuries to head, neck or back (see p. 37)
Broken limbs (see p. 39)
Allergic reaction to bee or wasp stings (see p. 48)
Poisoning (see p. 49)

! Bleeding can also occur inside the body. Although the blood loss is unseen, this is a life threatening situation. This can happen after a road accident or a fall (see chapter 'Severe bleeding' p. 30).

Step 3 *Seek help*

- Once you have evaluated the ill or injured person’s condition you can decide if help is needed urgently.
- An ambulance is the best way to transport ill or injured persons. If an ambulance can be obtained in a short time, it is best to call and wait.
- In case of road accidents, you should also consider seeking police help.
- Other types of transport can be used if no ambulance is available. See the box on transportation.

Step 4 *Give first aid*

Introduce yourself and explain what you are going to do. This will give the ill or injured person greater confidence in you. Always ask a person that is conscious or his family if you may help him. Try to give first aid to the person in a calm and controlled manner. We explain the exact procedures to follow in the chapters to come. Give priority to any life threatening conditions. First aid for minor conditions comes next.

When there are multiple ill or injured persons:

- give priority to persons in a life threatening situation;
- leave persons that only have minor injuries;
- leave persons that are dead.

Caution

Using alcohol for pain relief can be dangerous and should be avoided.

Transportation

- Move the ill or injured person with great care to the available means of transport. While en route: Drive carefully for the comfort of the person and for the safety of all.
- Try to keep the breathing passage open of persons that are unconscious. (see chapter 'Unconsciousness' p. 19).
- Try to support the neck and try not to twist the head, neck or body of persons with head, neck or back injury (see chapter 'Injury to head, neck, or back' p. 37).
- Ask bystanders to drive so that you can help the ill or injured person.
- Consider where to find the nearest facility that can provide help.
- If possible, encourage family or loved ones to acompany the ill or injured person.
- Try to protect the person from cold and heat.

If you need to get the ill or injured person *into the car*.

- Move the passenger seat as far back as possible and recline the seat backwards.
- Slide the person carefully in the car.
- Use the recovery position for persons that are unconscious.
- Stay with him until you reach medical help. Give first aid in accordance with the following chapters.

If there is *no medical care* in your area, plan ahead for transporting ill or injured persons:

- Make a list of numbers to call in case of an emergency.
- Make agreements with professional or private drivers.
- Motorcycles and bicycles can be made into ambulances too.
- Agree on signs to place on the road when someone needs emergency transport.

The community can set up a fund to pay for transport and care in case of emergencies.

Stress in an emergency

It is only normal to feel stress if you are suddenly faced with the need to give first aid in a real emergency. Try to bring your emotions under control before you proceed. Take a moment of your time to stand back from the situation and regain your calm. Do not set about the task too hastily and do not under any circumstances place your own safety at risk.

It is not always easy to process a traumatic event emotionally. It is not unusual for first aiders to experience difficulty when working through their emotions afterwards. Talk to your friends, family, fellow first aiders or religious leader. If you are still worried, talk to a professional and seek counselling.

Protecting from infection

When dealing with ill or injured persons it is important to keep the risk of infection between yourself and the ill or injured person to a minimum.

- If possible, wash your hands with soap and water before and after you take care of an ill or injured person. Alternatively you can also use ash that is no longer hot to wash your hands.
- Avoid direct contact with blood or body fluid.
- Always have gloves within reach, if you can. Wear shoes to protect your feet from infection.
- Use lots of clean water to rinse out any blood or other body fluid that splashes into your eyes or mouth, straight away.
- Dispose any soiled bandages carefully. Put it in a plastic bag or bin and then burn or burry it.
- Throw away used materials and clean up any blood spills because it can cause infection to others.
- Be very careful with sharp objects. They should be thrown away with care (e.g. in a box) so that they form no danger to others.
- Use clean drinking water or boiled and cooled water if a person needs to drink.
- Use rubber gloves if there is blood or other body fluids like urine or vomit. You can also use a clean plastic bag.

  ! If no gloves or plastic bags are available you can also direct the ill or injured person what he can do himself.
- Use a sticking plaster, bandage or clean cloth to protect any cuts, grazes, or wounds you may have your self. Infections may spread through breaks in your skin.

Caution

- If no gloves or plastic bag are available be very careful to avoid contact with blood. If you cannot ensure that, you can decide not to give help.
- Be careful with dirty or contaminated materials to treat ill or injured persons as they can pass on diseases from one person to another. You can sterilize material by placing it 10 minutes in boiling water or running it through a flame a few times.

For a full description of the technique "washing hands" the reader should consult the full African First Aid Materials.

Important

Seek medical help if you have been accidentally exposed to blood or body fluids. Certain medication may reduce your risk of infection.

Basic needs

Helping with stress

- Tell the ill or injured person your name and explain how you will help him. This will help to relax him.
- Listen to the person and show concern and kindness.
- Make him as comfortable as possible.
- If he is worried, tell him that it is normal to be afraid.
- If it is safe to do so, encourage family and loved ones to stay with him.
- Explain to the ill or injured person what has happened and what is going to happen.

Helping in case of cold or heat

Try to protect an ill or injured person from cold and heat.

- For cold: do not expose the person and use a blanket, coat or clothing to cover him. **weak**
- If the ill or injured person is outside in the sun, make a sunshade using an umbrella, blanket or a jacket. If this is not possible, use your own shadow to protect the ill or injured person.

Food or drink for injured persons

It is better not to give anything to eat or drink to a person that is:

- severely injured;
- feeling nauseous;
- becoming sleepy or falling unconscious.

This may create complications when the person needs an operation. **weak**

This does not apply to fever, malaria, pneumonia, diarrhoea or rash. See the corresponding chapters for more information.

Evidence that forms the basis for the recommendations

Dealing with an emergency

STEP 1 MAKE THE AREA SAFE

Evidence

- We did not find any relevant evidence, but it is common sense that safety is an absolute priority in first aid.

Additional information

- Traffic accidents are frequently associated with internal bleeding[27].
- In many cases no professional help is available to extricate persons from car wreckages. In those cases extrication is done by basic first responders. This sometimes leads to secondary injury when instruments like axes or machetes are used or when persons still entangled in the car are pulled out. [4;28]

STEP 2 EVALUATE THE CONDITION OF THE INJURED OR ILL PERSON

- The evidence and additional information are described in the following chapters.

STEP 3 SEEK HELP

Evidence

- We did not find any relevant evidence, but the recommendations reflect a common sense approach.

Additional information

- Self-treatment and cultural remedies from informal care providers occur in many situations[29-31] and often lead to a delay in obtaining medical care[28;32-35].
- Distance to medical care and transport problems form barriers to seeking medical care[35-38]. Transport often consists of a ride in private transport like taxis, other individual road users, or public transport[28;32]. Different types of community-owned and -managed emergency transport solutions have been effective[39]. This includes agreements with professional drivers; community-managed funds or prepayments for arranging emergency transport with private drivers; placing agreed signs along the road to ask for a lift to the hospital; locally manufactured bicycles with trailers or tricycles with a platform. The desire of family members to accompany the sick or injured person can influence the acceptability of the transport solution[39].
- Financial constraints can pose a barrier to seeking medical care[35-37;40]. Because of the costs, persons with multiple diseases sometimes receive medication for one disease only[41].
- Another barrier is how quality of medical care is perceived[21;35;36;38;40].

STEP 4 GIVE FIRST AID

- The evidence and additional information are described in the following chapters.

Protecting from infection

Evidence

- Washing hands using liquid soap and water is an effective method of hand hygiene to prevent respiratory infections[(42), B] and diarrhoea[((43), B]. The expert panel also considers handwashing as an effective component of infection control in general.
- Soap bars and ash appear to be equally effective in reducing bacterial counts on hands. Increasing the rubbing frequency and amount of water might decrease the bacterial counts.[((44;45), D]

Additional information

- Cross-infection through contact with blood or body fluids of the victim can be avoided by using gloves or another barrier (e.g. plastic bag). However, rubber gloves for self-protection are often not available in first aid situations. Many persons trained in first aid do not keep protective barriers within reach[4].
- Some cultural remedies such as topical application of cow dung, dirt or soil pose a high risk for infections[46].
- It is a challenge to make individuals wash their hands adequately[43].

Basic needs

HELPING WITH STRESS

Evidence

- Accupressure can reduce pain, anxiety, and heart rate in trauma victims[(47), B]. However, the expert panel does not recommend this technique because of local acceptability issues and the risk of causing harm in case of head or spinal injuries.
- Trauma victims appreciate self-help information on post-traumatic stress disorder, but it might not lead to better mental health outcomes[(48;49), C]. It appears that victims especially value information explaining that their reaction is typical for a person in a crisis[49].

Additional information

- Family members often wish to accompany the sick or injured person[39].

HELPING IN CASE OF COLD OR HEAT

Evidence

- Persons with hypothermia after trauma have worse outcomes than those without hypothermia[(50), C]. It appears that the body temperature continues to decrease even when blankets, cotton sheets, reflective blankets are used[(51), C].

Additional information

- Prevention of hypothermia is of importance in Africa, due to the long delays before reaching medical care[28].

FOOD OR DRINK FOR INJURED PERSONS

Evidence

- Drinking clear liquids until two hours before surgery is safe for most people[(52;53), B]. However, these findings cannot be generalised to emergency situations where victims are considered to be at high risk of regurgitation.

Additional information

- There can be serious delays in reaching medical help after traumatic injuries[28] or sudden illness[32].

Sudden illness

Facial weakness, arm weakness and speech problems

If a person suddenly has facial weakness, arm weakness or speech problems she might have had a stroke. This is a life-threatening situation. A stroke occurs when blood cannot reach a part of the brain. When a person suffers a stroke she can have difficulties doing certain actions.

If you think that someone is having a stroke you should ask the ill person to perform 3 simple actions and check for the following signs:

- Ask the ill person to smile or show you her teeth. Check whether her mouth is crooked or drooping at one corner.
- Ask the ill person to lift both arms. Check whether she can do this without one arm dropping or drifting. A stroke often causes one side of the body to become weak or even paralysed.
- Ask the ill person to repeat a simple sentence after you. Check whether she can speak clearly or if she has problems saying the words.

! A stroke is very likely if the ill person has difficulties with any of these actions. **strong**

What do you do?

1. Ask a bystander to seek help or to arrange for bringing the ill person to a medical care provider. Tell him to come back to you to confirm if help has been secured. The ill person urgently needs help. Shout or call for help if you are alone but do not leave the ill person.
2. If the person can sit up then make her sit upright. This helps the ill person to breathe. If the ill person can't sit, place her in the recovery position. **weak**
3. Comfort the ill person and explain what is happening to her. Tell the ill person to relax and rest. She should not try to do anything.
4. Arrange urgent transport to medical care yourself if you are alone.
5. Keep checking that the ill person is awake and breathing properly. In case the person becomes unconscious or is not breathing see chapter 'Unconsciousness' p. 19 or 'No breathing' p. 21.
6. Once actions to obtain help have been made, stay with the ill person until medical help is available.

Caution

Do not give food or drink to an ill person having a stroke. A person with a stroke is at risk of choking or vomiting. **weak**

Evidence that forms the basis for the recommendations

Facial weakness, arm weakness and speech problems

Evidence

- The face, arm, speech test appears to be a simple and adequate method for the initial evaluation of stroke[(54), A].
- Persons with stroke that have breathing difficulties and sit in a chair have a better oxygen saturation than those lying[(54), B].
- The evidence on aspirin for acute stroke shows a benefit for ischaemic stroke but potential harm for haemorrhagic stroke[(54;55), B]. Because during an acute stroke basic first responders cannot distinguish between the two types of stroke, the expert panel does not recommend aspirin.

Additional information

- Perceived causes of stroke include supernatural and biomedical explanations[21;24].
- Persons with stroke often seek plural treatment from healers, prophets or clinics[24]. The decision to seek treatment is often made by relatives, because the person is too ill to decide[21].
- Delayed help-seeking behaviour for potential stroke is a serious problem in African countries[32].

Chest discomfort

If someone complains of chest discomfort, it may be a sign that not enough blood is going to the heart. This is very serious and can lead to a heart attack. It is serious even when an ill person says that nothing is wrong.

weak

Suspect a heart attack if someone has the following symptoms:
- discomfort, pain, or tightness in the chest;
- pain spreading to shoulder, neck, jaw, arm or stomach;
- dizziness and fainting;
- sweating, difficulty breathing normally, nausea or vomiting.

These symptoms do not always happen all at once: they can happen very slowly.

What do you do?

1. Ask a bystander to seek help or to arrange for bringing the ill person to a medical care provider. Tell him to come back to you to confirm if help has been secured. The ill person urgently needs help. Shout or call for help if you are alone but do not leave the ill person.
2. Make the ill person comfortable. Make him rest and ask him not to move. Comfort the ill person and tell him what is happening.
3. Ask the person if he is taking medication and has taken it according to prescription. Do not delay getting formal medical attention.
4. If you have an aspirin, make the ill person chew an aspirin and swallow it with water afterwards. Tell him that this will help to get blood to the heart. strong

   ! Only give aspirin if the legislation in your country allows you to do this.
5. Arrange urgent transport to medical care yourself if you are alone.
6. Keep checking that the ill person is conscious and breathing properly. In case the person becomes unconscious or is not breathing see chapter 'Unconsciousness' p. 19 or 'No breathing' p. 21.
7. Once actions to obtain help have been made, stay with the ill person until medical help is available.

Evidence that forms the basis for the recommendations

Chest discomfort

Evidence

- A heart attack should be suspected in case of symptoms such as chest discomfort, pain radiating to arm, shoulder, neck, lower jaw or stomach; shortness of breath; sweating; dizziness; fainting; nausea; tendency to vomit[(56), C]. The presence of pain when palpating the chest is useful to rule out the chance of infarction[(56), C]. Asking basic first responders to palpate the chest for pain runs the risk of wrong conclusions being drawn, and persons with a heart attack not being recognised.
- Administration of aspirin reduces mortality and morbidity[(55), B].
- Chewing an aspirin tablet is the fastest way to absorb aspirin into the blood and to obtain an anti-platelet effect[(57), C].
- We did not find any evidence on which position is best for a heart attack.

Additional information

- Delayed help-seeking behaviour for potential heart infarction is a significant problem in African countries[(32)].
- Symptoms might evolve gradually, instead of abruptly as it is often presented[(58)]. It is important to explore locally how persons that have experienced a heart infarction describe their symptoms.

Choking

Infants and children often choke on swallowing foreign objects such as coins and small toys. Most adult cases of choking occur while eating. Since choking often occurs while eating there are usually people present. This means there is a good chance that someone will be able to give help quickly.

Someone may be choking if:

- they try to cough something up but it does not help;
- they cannot speak or make any sound;
- they put their hands on their throat;
- their lips and tongue turn blue;
- you can see the veins in their face and neck sticking out;
- the person becomes dizzy and then falls unconscious.

What do you do?

Ask the ill person: “Are you choking?”

If the ill person *can answer, cough or breathe*, then:

1. Ask the ill person to keep coughing.
2. Do not do anything else.
3. Stay with the ill person until he breathes normally again.

If the ill person *cannot speak, cough or breathe*:

1. You give 5 blows to the back, between the shoulder blades.
2. After each blow, you check if the object is still stuck in the breathing passage. Check by making eye contact after each blow. If the breathing passage is free (this means the ill person can speak, cough or breathe again), stop giving blows to the back.
3. If the object is still stuck you give 5 thrusts to the abdomen.If the breathing passage is free, stop giving abdominal thrusts.
4. If the ill person is still choking, you switch between 5 blows on the back and 5 abdominal thrusts.
5. If the ill person loses consciousness, you carefully place him on the ground.
6. Ask a bystander to seek help or to arrange for bringing the ill person to a medical care provider. Tell him to come back to you to confirm if help has been secured. The ill person urgently needs help. Do this yourself if you are alone.
7. You give 30 chest compressions and 2 rescue breaths.
8. Continue compressions and rescue breaths until medical help takes over or the ill person starts breathing normally or you become exhausted.

Caution

- Don't lose time.
- If the ill person’s chest does not rise during the first rescue breath do the following before attempting the second rescue breath:
  - Check the ill person’s mouth. Remove anything that obstructs the breathing passage.
  - Check that his head is tilted far enough back and that his chin is properly lifted.
  - Do not attempt more than two breaths each time before returning to the chest compressions.
- Abdominal thrusts can cause serious internal damage. Persons who have been given abdominal thrusts should be referred to a doctor.

For a full description of the techniques "back blows" and "abdominal thrusts" the reader should consult the full African First Aid Materials.

Evidence that forms the basis for the recommendations

Choking

We did not perform a systematic review for choking because evidence-based guidelines with instructions for basic first responders are available. Below we give an overview of the key findings of these guidelines[16].

- It has been shown that back blows, abdominal thrusts and chest thrusts may all be effective in relieving severe airway obstruction.
- Chest thrusts generate higher airway pressures than abdominal thrusts. Because chest thrusts are very comparable to chest compressions, a basic first responder should begin resuscitation if a person loses consciousness.

Fainting or unconsciousness

A person has lost consciousness if he does not react to your actions by opening his eyes or answering. Loss of consciousness causes the muscles to relax and this means that the tongue is also loose and can block the breathing passage by falling backwards in the throat.

In fainting, the loss of consciousness is usually brief. Fainting can be caused by many reasons such as emotional distress, tiredness, hunger, standing up for long times, a sudden change in body position, being a long time in hot environmental temperatures or specific medical conditions. Pregnant women, children and elderly can be more vulnerable to these causes.

Unconsciousness is a more serious type of loss of consciousness. Causes include head injury, heart arrest, stroke, or poisoning.

What do you do?

Talk loudly to the ill person. Tap him on the shoulders and ask if he is okay.

! Do not shake the person roughly as this may make his injuries worse.

If the ill person *responds*, then:

1. Do not try to change the position of the ill person's body. If there has been a head, neck, back, leg or arm injury, any movement may make the injury worse. Do not move him away from the scene unless he is in danger.
2. Try to find out what is wrong with the ill person.
3. Find medical help for the ill person if this is needed.
4. Keep checking the ill person to make sure that he is not getting worse.

If the ill person *does not respond*, then:

1. Shout for help. Do not leave the ill person if you are alone. Ask a bystander to seek help or to arrange for bringing the person to a medical care provider. Tell him to come back to you to confirm if help has been secured. The person urgently needs help.
2. You must unblock the breathing passage:
   - Gently roll the ill person over on to his back.
   - Carefully tilt the head back and lift the chin up with your hand on the bony part of the chin.
   - Do not put your hand on the soft part under the chin to do this.
   - This simple action lifts the tongue from the back of the throat.
3. The ill person’s airway must be kept open while you check for breathing:
   - Check that the ill person’s chest is going up and down.
   - Put your ear to the ill person’s mouth to listen for breathing.
   - Put your cheek close to the mouth to feel for breathing.

   ! Do not spend more than 10 seconds looking, listening and feeling for an ill person’s breathing.

If the ill person is *not breathing*:

You give first aid as in chapter 'No breathing' p. 21.

If the ill person is *breathing normally* but does *not react*:

1. You should roll the ill person into the recovery position and find emergency medical help.
2. Once actions to obtain help have been made, stay with the ill person until medical help is available.
3. Keep checking that the ill person is breathing without difficulty.

Caution

In the first few minutes after a heart arrest it often appears as if the ill person is trying to breathe. It can appear as if the casualty is barely breathing or is taking infrequent noisy gasps. In fact, they are the last 'gasps' of a body in the throes of death. You should not confuse this with normal breathing. If you are not sure if the ill person is breathing normally then you do the same as for a person who has stopped breathing. Keep checking that the ill person is breathing without difficulty.

Unblocking the breathing passage takes priority over concerns about a potential spinal injury. Unless you can clearly see that the person is breathing normally an unconscious person must be turned onto his back to unblock the breathing passage and to check breathing.

When a person needs to be put in the recovery position, keeping the airways open takes priority over potential spinal injury. If possible, support the person's neck while turning him into the recovery position.

For a full description of the technique "recovery position" the reader should consult the full African First Aid Materials.

Evidence that forms the basis for the recommendations

Unconsciousness

We did not perform a systematic review for unconsciousness because evidence-based guidelines with instructions for basic first responders are available. Below we give an overview of the key findings of these guidelines[16].

- In an unconscious person, the muscles are relaxed. This causes the tongue to obstruct the airway. The risk can be eliminated by carefully tilting the head back and lifting the chin.
- In the first few minutes after cardiac arrest it often appears as if the person is trying to breathe. It can appear as if the person is barely breathing or is taking infrequent noisy gasps. First responders should be taught not to confuse this with normal breathing and to start resuscitation.
- Feeling the carotid pulse is an inaccurate method of establishing the absence of circulation and should only be applied by professional healthcare providers experienced in the technique.

No breathing

A person can only survive a few minutes without breathing and a beating heart. If you find an ill person who is not breathing, you can increase his chances to stay alive by pushing hard and fast in the middle of the ill person's chest and giving rescue breaths.

What do you do?

If there is *no reaction* from the ill person and he is *not breathing normally*, you should:

1. Ask a bystander to seek help or to arrange for bringing the ill person to a medical care provider. The ill person urgently needs help. Do this yourself if you are alone.
2. Start pushing down hard and fast in the centre of the chest: do this 30 times without stopping.
3. Give 2 rescue breaths. This means breathing into the ill person's mouth.
   - ! If for some reason you cannot or do not want to give rescue breaths you can just continue to push down on the chest.
4. Push down 30 times hard and fast on the chest again.
5. Give 2 rescue breaths again.
6. Do not interrupt resuscitation until:
   - professional help arrives and takes over; or
   - the victim starts to wake up: to move, opens eyes and to breathe normally; or
   - you become exhausted.

- For a baby, only use two fingers to push down on the chest, otherwise you might hurt it more. Place a baby on a firm surface.
- For a child, use one or two hands depending on the size of the child and your own strength.
- For babies and children, you should push the breastbone for at least one third of the depth of the chest.
- It takes less air to give rescue breaths to babies or children. Check that the chest rises.
- If it rises, then you have blown enough air in.

In case of *drowning*:

- Remove the injured person rapidly and safely from the water, but do not place yourself in danger.
- Try to throw a rope or something that floats to hold onto to injured persons that are conscious.
- Do not try to remove water from the lungs.
- Start chest compressions and rescue breaths immediately.
- Cover the injured person with a coat or a blanket to keep warm.

For a full description of the technique "chest compressions" the reader should consult the full African First Aid Materials.

If there are a few trained rescuers present, it is best to alternate with each other during resuscitation. Chest compressions are tiring to administer. The quality of the chest compressions often deteriorates after a few minutes. The rescuer does not always realise this. To ensure the quality of chest compressions, the rescuers should switch every two minutes. The switch should preferably be made after giving two ventilations.

- The first rescuer resuscitates for two minutes (chest compressions and ventilations).
- Another rescuer takes over and resuscitates for a further two minutes (chest compressions and ventilations). Then switch again.
- The switch should happen with minimal interruption, and as quickly as possible.

Evidence that forms the basis for the recommendations

No breathing

We did not perform a systematic review for this topic because evidence-based guidelines with instructions for basic first responders are available. Below we give an overview of the key findings of these guidelines. Pushing hard and fast in the middle of the ill person's chest, with minimal interruption, is the most vital part of resuscitation[59;60].

- Chest compression ensures a small but crucial supply of blood to the heart and to the brain[16].
- The provision of chest compressions with ventilations is the preferred option for trained first responders [16]. Chest compression only is a reasonable alternative for trained first responders if they cannot or do not want to give rescue breaths [16].
- Resuscitation procedures are difficult for basic first responders. It is important that instructions are simple to learn and to remember.
- The chances of survival after resuscitation are small, but increase with immediate resuscitation[16].
- There is no evidence that water acts as an obstructive foreign body, so no time should be wasted trying to remove it[61].
- There is no evidence of the effect of prophylactic antibiotics in near-drowning incidents[62].

Fever

A fever can be a sign of serious illness. Any person with fever needs medical attention to determine the cause. Fevers caused by malaria or pneumonia* can be very dangerous if they are left untreated and can lead to death.*

** Malaria is a disease that is spread by mosquitos. An ill person with fever who lives in, or has visited, a malaria region may have malaria.*
** Pneumonia is an infection of the lungs.*

What do you do?

1. Wash your hands before taking care of an ill person. Use soap to wash your hands or alternatively you can also use ash. **strong**
2. Find out how high the ill person's temperature is:
   - Use a thermometer in the armpit, if available. The ill person has a fever if his temperature is higher than 37,5°C.
   - If you do not have a thermometer, and the person feels hot to touch, it is probably a fever. **weak**
3. Seek medical help to find out the cause of the fever.
4. Someone with fever needs to rest and drink lots of fluids to stop dehydration:
   - Give the ill person more to drink if the colour of urine is dark and the ill person does not urinate often.
   - Breast-fed babies: continue to breast-feed but more frequently than usual.
   - Bottle-fed babies: continue with normal feeds and give extra rehydration drinks.
5. Think about how the ill person is dressed. Dressing too warm can increase the fever, dressing too lightly can cause shivering which will deplete the body energy.
6. Use lukewarm water to sponge the ill person unless it upset him or causes shivering.
   ! Do not use cold water as this can make the body react by heating up more. **weak**
7. If an ill person is suffering, give an anti-fever medication if allowed in your country. **strong**
8. If the ill person has a fit (he suddenly shakes fast and uncontrollably), give first aid for fits (see chapter 'Fits' p. 26).
9. Wash your hands after taking care of an ill person. Use soap to wash your hands or alternatively you can also use ash. **strong**
10. Keep checking the ill person day and night: get up two or three times in the night to check.

When to seek medical help

Fever can be a sign of serious illness. Any person with fever needs medical attention to determine the cause. Medical attention is especially important for babies, children and pregnant women.

Seek emergency medical help if the ill person:
- cannot take medication;
- has fits;
- is very sleepy, difficult to wake up, or confused;
- has a headache;
- keeps vomiting;
- cannot drink, urinates less and the colour of the urine darkens, sunken eyes, an ill child cries without tears, mouth is dry;
- cannot stand up or sit up;
- is a baby and is too weak to be carried;

- has fast breathing:
  - child up to 12 months: more than 50 breaths/minute.
  - child more than 12 months: more than 40 breaths/minute.
- has difficulty breathing, such as the chest heaving, nostrils flaring or chest indrawing;
- has a whistling noise when breathing;
- is bleeding spontaneously.

If an ill person must travel for help, keep giving him sips of drink on the way there.

Caution

- Only give anti-fever medication if the legislation in your country allows you to do this.
- Anti-fever medication and herbal remedies may bring relief, but do not treat the cause of the illness.
- Keep the person away from any smoke, including cooking fires and cigarettes.

Important

When medication is prescribed:
The ill person must finish the whole course of medicine. If it is not finished, then the person is not cured and the disease may come back.

- There is an exact amount of medicine to give according to the ill person’s age and weight.
- You must stick to this dosage.
- When giving medicine to an ill child, make sure he is calm. An ill child who is crying will not swallow the medicine.
- If an ill person vomits less than 30 minutes after taking the medicine, give the medicine again.

Avoid purchasing substandard medication or using medicines that

- have past their expiry date;
- have been exposed to direct sunlight;
- have been wet.

Evidence that forms the basis for the recommendations

Fever

Evidence

- Detection of fever by palpation, is more helpful in ruling out fever than confirming fever[(63;64), D].
- We did not find any evidence that compared touch with the back of the hand versus the palm for detecting fever.
- Paracetamol reduces fever but does not lead to faster healing[(65), B].
- It is unclear if physical methods alone have an antipyretic effect. Physical methods combined with paracetamol further reduce fever. Physical methods cause a certain discomfort for the patient.[(66), C]
- The presence of fever appears to be an acceptable indicator of malaria, both in areas of low and high malaria prevalence[(67), D].
- Rapid breathing appears to be a useful factor for identifying pneumonia in children and can be identified by lay health workers who count the respiratory rate[(20;68-70), D]. No combination of signs has been shown to give absolute certainty in diagnosing pneumonia[71;72].

Additional information

- Measuring the temperature in the armpit is easy and avoids the risk of rectal damage and faecal contamination.[73]
- In a person with fever, dehydration can develop unnoticed. The signs of dehydration are often mistakenly linked to discomfort due to the illness itself. Checking urine colour and output is potentially a simple way to check dehydration[71].
- Fever or malaria are often perceived to be the result of malicious intent, overexposure to the sun, or poor hygiene[25;74;75].
- The risk of malaria is higher among young children and pregnant women[67].
- Malaria is often managed in the home. However, self-treatment runs the risk of using the wrong medication or incorrect dosage[29-31]. The impact of home-based management of malaria is unclear[(76-79), D]. Serious problems are associated with substandard medication circulating in the African region[67].
- Partial treatments should not be given even when patients are considered to be semi-immune or the diagnosis is uncertain[67].
- Symptoms of malaria and pneumonia overlap, and lay caregivers often consider cases of pneumonia to be malaria. The perceived risk of getting malaria is high, and for pneumonia low.[80]
- Mistreatment of pneumonia with antimalarials is common[30;80-82].

Fits

An ill person has a fit if she suddenly shakes uncontrollably. It is different to normal shivering and trembling. It may manifest all limbs or just a single limb. The person having a fit may also urinate or defecate without control. A fit can be caused by a high fever, malaria, epilepsy, alcoholism or drugs. Epilepsy is a common illness caused by a problem in the brain.

What do you do?

1. Remove objects that could hurt the person or move her to a safe place.
2. If possible, put during the fit something soft under her head if the ill person lies on the ground.
3. Make sure she can breathe freely by loosening tight clothing around her neck, such as a collar or tie.
4. When the fit stops, place her in the recovery position. This will keep her breathing passage open. It will also prevent vomit from entering her lungs (see chapter 'Unconsciousness' p. 19).
5. Stay and talk calmly with her until she feels better.
6. If the fit was caused by a high fever, give first aid for fever (see chapter 'Fever' p. 23).

Caution

DO NOT try to hold the ill person down or put anything in her mouth:

- an ill person cannot swallow her own tongue during a fit;
- an ill person might bite her own tongue but this normally heals in a few days;
- an object or a hand placed in the mouth of someone having a fit is dangerous for the ill person and yourself.

When to seek medical help

A fit can be a sign of a serious illness. Any person with fits needs medical attention.
Medical attention is especially important if:

- it is the ill person's first fit;
- the fit lasts longer than five minutes
- there is more than one fit and the ill person does not wake up in between;
- there is a high fever;
- the ill person has hurt himself.
- If the ill person is alcoholic or under the influence of drugs: talk to her kindly and without judging.

Evidence that forms the basis for the recommendations

Fits

Evidence

- There is no evidence that paracetamol prevents febrile fits[83;84].

Additional information

- Forced drinking or inserting fingers or a spoon into the mouth of a person with fits often results in injuries[85].
- It is sometimes believed that fits are contagious or have supernatural causes[86-89].
- Intentionally burning the feet sometimes occurs as a cultural remedy for treatment of epilepsy[46]. This practice is discouraged by the panel.

Diarrhoea

Diarrhoea is usually caused by an infection. A person can catch this infection by:

- *not washing his hands;*
- *touching faeces;*
- *eating unsafe food, such as fish that was caught in polluted waters;*
- *drinking bad water;*
- *preparing food with bad water;*
- *food that has not been kept cold or has gone bad.*

Diarrhoea causes dehydration as too much water and nutrition leaves the body. If an ill person does not receive help, he can die. Babies and children are most at risk.

What do you do?

1. Wash your hands before and after taking care of an ill person. Use soap to wash your hands or alternatively you can also use ash. You could pass on a disease to him and also catch his infection. **strong**
2. Prevent dehydration: at the first sign of diarrhoea, give the ill person plenty to drink.
   - There are special rehydration drinks that you can buy in sachets from the pharmacy. **strong**
   - Alternatively you can also prepare a rehydration drink yourself: **weak**
     - mix two fistfuls of maize flour (60g) with 1l of water;
     - add two pinches of salt and mix it well.
     - Stir continuously until it boils.
     - Add a bit of water if the solution is too thick to drink.

   ! If you cannot buy or prepare the rehydration drinks, then give clean drinking water as the main drink.
3. Each time an ill person passes diarrhoea, he must drink to replace what he has lost.
   - For children under 2 years old: between a quarter and half a large cup of fluid (50-100 ml).
   - For children from 2 to 10 years old: between half and a full large cup (100-200 ml).
   - For older children and adults: at least 1 large cup (200 ml).
4. If the ill person vomits, wait for 5-10 minutes before you give another drink, then use a spoon to give the drink more slowly.
5. Children should eat as normally as possible:
   - Breast-fed babies: continue to breast-feed but more frequently than usual.
   - Bottle-fed babies: continue with normal feeds and give extra rehydration drinks.
   - Older children and adults: eat as soon as they feel like it.
6. Wash your hands after taking care. Use soap to wash your hands or alternatively you can also use ash. **strong**
7. Try to obtain zinc tablets. This will help to fight the diarrhoea. **strong**
8. If the person also has fever, see chapter 'Fever' p. 23. of an ill person.

Caution

- When mixing the rehydration drink or baby formula, make sure you use clean drinking water or boiled and cooled water.
- Do not store unused drinks but throw them away.
- An ill person with diarrhoea does not normally need antibiotics, unless a qualified health worker has told you so.

When to seek medical help

Persons with diarrhoea may dehydrate. If an ill person does not receive adequate help, he can die.

Seek medical help if the ill person becomes more ill or if you see any of the following signs:
- very bad diarrhoea, blood in the diarrhoea;
- great sleepiness, difficulty in waking up, confusion;
- vomiting everything;
- the ill person urinates less and the colour of the urine darkens, sunken eyes, a child cries without tears, mouth is dry;
- the ill person is not drinking;
- fits;
- breathing seems wrong;
- the diarrhoea is not getting better after 2 days.

If an ill person also has a fever (see chapter 'Fever' p. 23).

If an ill person must travel for help, keep giving him sips of drink on the way there.

Evidence that forms the basis for the recommendations

Diarrhoea

Evidence

- Children with mild diarrhoea can drink undiluted milk[(90), B]. Giving lactose-free feeds is generally not required[(90), B].
- Oral rehydration solution (ORS) with a reduced sodium concentration is more effective than standard WHO ORS (90 mmol/l of sodium and 111 mmol/l of glucose and a total osmolarity of 311 mmol/l).[(91), A].
- Manually prepared salt and sugar solutions has led to concern over safety. A home-based study provides evidence that preparing maize ORS might be safer than glucose ORS[(92), C].
- The research on ORS focuses on the treatment of dehydration with ORS. However, the role of ORS in preventing dehydration is unclear[(93)].
- Zinc deficiency occurs among many children in developing countries, and zinc supplementation reduces the duration of and recovery from acute diarrhoea in children[(91), B].
- Delayed skin recoloration time and the skin fold are useful for healthcare professionals when determining dehydration[(94), D]. However, slow skin pinch has a poor outcome for detecting dehydration when performed by trained community health workers[(20), D]. The absence of dry mucous membranes is the most useful sign for healthcare professionals to rule out dehydration[(94), D]. Signs related to the inability to drink, vomiting everything, lethargic state, diarrhoea, being restless or irritable have a moderate sensitivity when performed by trained community health workers[(20), D]. No sign of dehydration is sufficient on its own to evaluate the dehydration status of a child[(94), D].

Additional information

- It is sometimes believed that diarrhoea in infants is caused by bad mother's milk, teething or supernatural causes. Gouging and extracting tooth buds or teeth are sometimes done as a cultural treatment[(95)]. This can lead to severe bleeding or sepsis from non-sterile instruments and affects the dentition. Rubbing herbs on the gingiva is a non-invasive method, but might delay medical care[(96)].
- Low home-based use of commercial ORS has been reported. One barrier is that ORS does not treat diarrhoea itself, which does not provide enough incentive to buy the product. Another reason for low use of ORS is the difficulty in obtaining safe water[(22)].
- Antibiotics of unknown identity and quality are sometimes used for diarrhoea[(22)].
- If both fever and diarrhoea occur, lay caregivers often consider treatment of malaria only[(22)].

Rash

A child with a fever and a rash may have measles if it goes together with any of the following signs: cough, runny nose or red eyes.

A rash can be difficult to see on dark skin: look for roughness on the skin.

What do you do?

1. Wash your hands before taking care of an ill person. Use soap to wash your hands or alternatively you can also use ash. **strong**
2. A child with a fever and rash should be kept away from other children, especially babies.
3. If the child lives in a malaria region, the child should be treated for measles and malaria. Give first aid for fever (see chapter 'Fever' p. 23).
4. Wash your hands after taking care of an ill person. Use soap to wash your hands or alternatively you can also use ash. **strong**

When to seek medical help

Measles can cause death and can cause other infections. Sometimes measles can lead to complications, such as: malnutrition, blindness, deafness, lung disease, brain damage.

Seek medical help if you see any of the following signs:

- the child is not drinking;
- great sleepiness, the child is difficult to wake up;
- vomiting everything he takes in, diarrhoea, dehydration;
- fast breathing;
- fits;
- the child cannot bear light;
- earache or pus coming from the ear;
- eye infection;
- sores or open lesions in the mouth;
- spontaneous bleeding or small spots of blood leakage in the skin.

If an ill person must travel for help, keep giving him sips of water or liquid on the way there.

Evidence that forms the basis for the recommendations

Rash

Evidence

- Trained lay health workers can effectively diagnose measles based on the identification of fever and rash and at least one of the following: cough, runny nose, red eyes[(70), D].
- Routinely giving antibiotic prophylaxis to children with measles has little or no effect on the occurrence of pneumonia or mortality[(97), C].

Additional information

- Cultural practices for measles include rolling the undressed child in ash and rubbing the child with a mixture of leaves until the rash is crushed and opened. Juice from bark of the aloe tree and goat milk are sometimes applied to the eyes.[98] This practice is discouraged by the panel.

Injuries

Severe bleeding

A person who has an open wound which is bleeding severely is in a life-threatening situation and immediate help is needed. The blood spurts or continues to flow from the wound.

What do you do?

1. Ask a bystander to seek help or to arrange for bringing the injured person to a medical care provider. Tell him to come back to you to confirm if help has been secured. The injured person urgently needs help. Shout or call for help if you are alone but do not leave the injured person.
2. Check if there are no objects stuck in the wound. If there is an object in the wound do not remove it (see chapter 'Wounds with bullets or objects in a cut' p. 32).
3. If the injured person is conscious, ask him to press on the wound himself.
4. Help the person to lie down and comfort him.
5. Try not to touch the person's blood. Put on rubber gloves if available. You can also use a clean plastic bag. Use a clean cloth to press on the wound. **strong**
6. Press on the wound with both hands. If the wound keeps bleeding, press harder on the wound. Keep pressing on the wound until help arrives. **weak**
7. You can also wrap a bandage around the wound to slow down the bleeding. If you do not have a bandage, you can also use clean clothing and tape for this.
8. Make sure the bandage is firm enough so it stops the bleeding but doesn't cut off all the blood flow. If the part of the body below the bandage changes colour or is swelling or the injured person says he is losing any feeling there, loosen the bandage a little but do not remove it.
   If the blood flow to a limb is stopped an injured person can loose his limb.
   ! If the bandage becomes soaked in blood, do not remove it. Add another one on top.
9. Keep the injured person warm by taking off wet clothing, covering him with a blanket or other covering, but do not overheat the injured person. Keeping the person warm is important to delay the onset of shock. **weak**
10. Arrange urgent transport to medical care yourself if you are alone.
11. Stay with the person until medical help is available. Once actions to obtain help have been made, keep checking that the person is conscious and breathing properly.
12. Wash your hands after giving first aid. Use soap to wash your hands or alternatively you can also use ash. **strong**

Bleeding can also occur inside the body. This can happen after a road accident or a fall from a great height. Although the blood loss is unseen, this is a life threatening situation.

Suspect bleeding inside the body if the injured person:
- is losing blood from body openings;
- is breathing rapidly;
- has a cold and clammy skin that is pale or turns blue;
- is behaving in an irritated or unusual way;
- becomes sleepy or falls unconscious.

Keep the injured person warm and seek emergency medical help immediately. **weak**

Caution

- Do not raise the injured persons legs. The effect is very limited and might even cause harm. **weak**
- Do not try to stop the blood flow in a limb with a tourniquet or very tight bandages. If the blood flow to a limb is stopped, the limb can be lost. **weak**

Evidence that forms the basis for the recommendations

Severe bleeding

Evidence

- There is evidence that direct compression on the artery prevents bleeding[(1), B]. There is no evidence about the effectiveness of indirect pressure on pressure points and elevation for severe bleeding.
- Improvised tourniquets have a high morbidity rate and should not be used as a first aid measure for bleeding[((99), D)].
- Hypothermia due to severe bleeding may further increase blood loss and cause complications[((51), C)].
- Raising the legs has been a custom first aid practice for shock. However, the evidence shows little and transient effects on haemodynamics and there is a risk of harmful effects from its use[((100), C)]. Therefore the panel recommends that it is better not to raise the legs.

Wounds with bullets or objects in a cut

Wounds with bullets, knives, arrows or pieces of glass can lead to severe bleeding and infection. Injuries to structures under the skin may cause loss of feeling or problems to move the body part. You should not try to remove any object because this will usually cause more bleeding and harm.

Even if you cannot see an object, there may be something stuck in the wound if:

- the injured person feels pain in a specific area;
- the injured person has a painful lump;
- the injured person has the feeling that something is in the wound;
- there is a discoloured area.

What do you do?

1. Ask a bystander to seek help or to arrange for bringing the injured person to a medical care provider. Tell him to come back to you to confirm if help has been secured. The injured person urgently needs help. Shout or call for help if you are alone but do not leave the injured person.
2. Try not to touch the person's blood. Put on rubber gloves if available. You can also use a clean plastic bag. **strong**
3. If there is an object stuck in the wound, do not remove it because this can cause further damage or bleeding. Check if the object caused an additional exit wound if it passed through. Try to stop or slow down the bleeding. Be careful not to push the object deeper (see chapter 'Severe bleeding' p. 30).
4. Use sterile gauze to cover the wound if available, or use a clean dry cloth.
5. Try to stop the protruding object from moving with bulky material and bandages. Build up padding around the object until you can bandage over it without pressing down.
6. Bandage the material above and below the object.
7. Take off jewels or anything else in the area of the wound that may cut off blood flow because of swelling.
8. Arrange urgent transport to medical care yourself if you are alone.
9. Once actions to obtain help have been made, stay with the injured person until medical help is available.
10. Keep checking that the injured person is conscious and breathing properly.
11. Wash your hands after giving first aid. Use soap to wash your hands or alternatively you can also use ash. **strong**

Evidence that forms the basis for the recommendations

Wounds with bullets or objects in a cut

Evidence

- We did not find any relevant evidence.

Snake, scorpion or spider bite or sting

The effects of a bite are different according to the type of venomous animal.

Depending on the species there can be:

- bleeding, swelling, bruising;
- numbness, weakness, confusion, affected vision;
- heart arrest or difficulty breathing.

What do you do?

What do you do for *a snake bite*?

1. Ask a bystander to seek help or to arrange for bringing the injured person to a medical care provider. Tell him to come back to you to confirm if help has been secured. The injured person urgently needs help. Shout or call for help if you are alone but do not leave the injured person.
2. If safe to do so, check what type of animal it is, but do not try to catch it. If possible write down the features of the snake.
3. Watch the injured person for a change in his condition. Evaluating if a snake is poisonous or not is difficult. It is best to assume that the snake is poisonous.
4. Help the injured person to lie down and tell her not to move. Offer comfort and keep her calm. This will slow down the venom.
5. Try not to touch the person's blood. Put on rubber gloves if available. You can also use a clean plastic bag. **strong**
6. Take off any rings, watches or tight clothing that may cut off blood flow because of swelling. Be careful not to move the limb.
7. If venom gets in the eyes, rinse them for 15 to 20 minutes with water, from the nose outwards.
8. If the bite is in the leg: immobilise the leg by bandaging it to the other leg. **weak**
   - Gently bring the good leg to the bitten leg.
   - Use a stick to splint the limb and bandage it in place.
   - If the bite is on the arms, tell the injured person to immobilise her own arm by holding it close to her body until she obtains medical care.
9. Arrange urgent transport to medical care yourself if you are alone.
10. Once actions to obtain help have been made, stay with the injured person until medical help is available.
11. Keep checking that the injured person is conscious and breathing properly.
12. Wash your hands after giving first aid. Use soap to wash your hands or alternatively you can also use ash. **strong**

Caution

- Do not try to catch the snake.
- Do not try to suck or cut the venom out or do not rub herbs on as this will not help and can harm the person more.
- Tell the injured person not to move and to keep his limb very still. This will slow down the venom.

What do you do?

What do you do for *a spider bite or scorpion sting*?

1. Put on rubber gloves if available. You can also use a clean plastic bag. **strong**
2. Wash or wipe away any venom
3. Use ice, if you have it, to cool the bite or sting:
   - Wrap the ice in a cloth or a towel so it does not touch the skin directly.
   - If you do not have ice, use cold water.
   - Do not cool for more than 20 minutes at a time.
4. Find medical help if the pain does not decrease.

Evidence that forms the basis for the recommendations

Snake, scorpion or spider bites

Evidence

- The application of an elastic bandage combined with immobilisation is an often recommended first aid technique for snake bites. However, it appears to be difficult to apply the elastic bandage at the correct pressure[(101;102), C]. The elastic bandage should be applied tightly, but applying it too tightly or not tightly enough is ineffective and may worsen the injured person's condition[(103), D]. Applying an elastic bandage has a lower efficacy than applying an elastic bandage over a firm cloth pad[(104;105), C]. However, using a firm cloth pad runs the risk of creating an arterial tourniquet.
- One study indicates that immobilisation can be taught to basic first responders[(102), D]. However, a field study indicates that after receiving the instruction to immobilise limbs with snake bites, this was only done properly in a minority of cases[(106), D].
- Because elastic bandages and the firm cloth pad are difficult to apply adequately and may harm the injured person, the panel decided to limit the recommendation to immobilisation of the limb only.
- We found no evidence on spider or scorpion bites.

Additional information

- A wide variety of remedies are used for snake bites[(34)]. Suction only removes a very small amount of venom, and it may make the injury worse[(61)]. Incision, excision, heat, ice, cryotherapy, poultices, topical chemicals or herbals, alcohol or stimulants offer no benefit and may worsen outcomes.
- Apart from a study on animals[(107)], we did not find any studies on the effectiveness of the black stone or snake stone.
- The availability of anti-venom serum can be problematic in some African countries(34;108).
- Snake bites can lead to tetanus[(109)]. Fake or expired tetanus toxoids without potency are possibly being sold in pharmacies[(110)].

Burns

Burns can be a serious threat if they are not managed properly.

- With superficial burns the skin is red, slightly swollen and painful.
- If the burn is deeper you will also see blisters. These burns are extremely painful.
- If the deepest layer of the skin is burned there is usually no pain in the wound itself, because the nerves in this area have also been destroyed. The burn can look black, parchment-like or white and is dry. However the skin around the wound, which is often less deeply burned, is painful.

What do you do?

1. Ask a bystander to seek help or to arrange for bringing the injured person to a medical care provider. Tell him to come back to you to confirm if help has been secured. The injured person urgently needs help. Shout or call for help if you are alone but do not leave the injured person.
2. If clothing is on fire, you can douse it with water, wrap the injured person in a heavy blanket or make the injured person roll on the ground. Stop the injured person from running around.
3. Use clean water to cool the burn, if you can, otherwise any water. Pour water on the burn for 15-20 minutes, or until it stops hurting. Cooling prevents a burn from going deeper by taking heat away from the skin and reduces pain.
4. Put on rubber gloves. You can also use a clean plastic bag. **strong**
5. Remove any clothing and jewellery that is not stuck to the skin.
6. You can put liquid honey on the burn. This prevents infection and helps the wound to heal. Do not use warm or hot honey.
   Alternatively you can also use aloe vera. **weak**
7. Dress the burn with a wet wound dressing. Avoid dressings that may stick to the burn.
8. Bandage the dressing to the wound.
9. Wash your hands after giving first aid. Use soap to wash your hands or alternatively you can also use ash. **strong**
10. Change dressings once a day. When you change the dressing, make sure the old one comes off without sticking to the wound and damaging it: soak it in drinkable water first. **weak**

Caution

- An injured person can become hypothermic if he has large burns and needs to be kept warm:
  - Try not to use very cold water for cooling the burn.
  - Protect the injured person from the wind and wrap him in blankets.
- Leave blisters intact to prevent infection. **weak**
- Do not use vaseline for burns. Vaseline is not sterile and can cause infection.
- Keep flies and other insects away from the wound to prevent infection.

When to seek medical help

Minor burns can be safely managed at home. Seek medical help straight away if:

- the injured person is under 5 years old or over 65 years old;
- the burn is on the face, ears, hands, feet, the sexual organs or joints;
- the burn circles the entire limb, body or neck;
- the burn is equal or larger than the injured persons hand size;
- the burn looks black, white, papery, hard and dry;
- the injured person has no sense of feeling in the wound itself;
- the burns were caused by electricity, chemicals or high pressure steam;
- the injured person has inhaled flames or heat, or breathed in a lot of smoke.
- clothing or jewellery is stuck to the skin.

The injured person should seek medical help if it is more than 10 years since his last tetanus injection or if there is any doubt about when the injured person last had a tetanus injection. It is very safe to get a tetanus injection.

The injured person should seek medical care if in the days after, the burn smells bad, is soaked with pus or if he gets a fever.

Evidence that forms the basis for the recommendations

Burns

Evidence

- Suggestions for the duration of cooling range from 10 to 30 minutes, but the evidence for the optimum length is inconclusive[(111)].
- Treating burns with Aloe Vera[((112), C)] or honey[((113), C)] may reduce the healing time. Fresh Aloe Vera holds the risk of being contaminated and can cause irritation. Experts comment that there are differences in the antibacteriological activity of different kinds of honey[(114)].
- Sugar, papaya, and fatty acids are sometimes used as home remedies for wounds. There is insufficient evidence to draw any conclusions about their effectiveness[(115)].
- Changing dressings for burns once a day versus twice a day does not increase infection, causes less pain, and reduces the cost of dressings[((116), D)].
- Leaving blisters intact may result in fewer infected burns. Pain may be worse in blisters that are deroofed compared with those that are aspirated[((111), C)].

Additional information

- When cooling, hypothermia in the person must be prevented[(1)]. Prevention of hypothermia is of importance in Africa, due to long delays before reaching medical care.
- Many persons with burns never get medical care. Seeking help from informal care providers often creates a delay in obtaining medical care[(46)].
- Harmful remedies include topical application of cow dung, dirt or soil. Their use as a cultural remedy must be discouraged[(46)].
- Commercially available Vaseline describes on the bottle its indication for minor burns and is sometimes used as such. Vaseline is sometimes used as a home remedy for burns. However, Vaseline is occlusive, non-sterile and may lead to infection[(117)].
- Gauze dressings that stick to the wound can be removed with prolonged soaking[(118)].
- Sterilised banana leaves[((119), C)] or boiled potato peels[((119-122), C)] might be an alternative means of dressing burns. The panel does not recommend this type of dressing since it is not possible to sterilise such dressings without special equipment.
- The total body area that is burned, depth of the injury, age, and presence of other conditions determine the prospect of recovery[(111)].
- Tetanus immunisation is needed for burns but fake or expired tetanus toxoids without potency are possibly being sold in pharmacies[(110)].

Injury to head, neck, or back

Injuries to head, neck, or back can be very serious and should always be managed with caution.

Suspect a serious head or back injury if the person:
- has had a hard blow through a road accident or from falling from a height greater than standing height;
- becomes sleepy or falls unconscious;
- does not remember exactly what just happened;
- has a terrible headache that does not go away;
- is feeling sick to her stomach or is vomiting;
- is behaving in an irritated or unusual way;
- has a fit;
- has serious wounds or injuries on his head;
- complains that she feels numbness or a tingling feeling;
- has serious injuries on the legs and does not complain about the pain;
- feels pain or tenderness in the neck or back.

It is hard to know in what way an injured person is injured if the person
- is agitated;
- is in great pain;
- is drunk;
- is a child;
- is older than 65 years.

Seek medical help if you feel unsure about the injury.

What do you do?

1. Ask a bystander to seek help or to arrange for bringing the injured person to a medical care provider. Tell him to come back to you to confirm if help has been secured. The injured person urgently needs help. Shout or call for help if you are alone but do not leave the injured person.
2. If the injured person is unconscious (see chapter 'Unconsciousness' p. 19), you should make sure that she is breathing freely. If the injured person is conscious, calm her and ask her not to move.
3. To keep the head still, place your hands or tightly folded clothing on each side of the injured person's head. Only keep the head and neck still if the injured person will let you.
   ! If the injured person is upset or excited, do not force her to have her neck immobilised.
4. Keep the injured person warm but do not overheat her. **weak**
5. Arrange urgent transport to medical care yourself if you are alone.
6. Keep checking that the injured person is conscious and breathing properly.
7. Once actions to obtain help have been made, stay with the injured person until medical help is available.

Caution

- Do not move the injured person unless she is in more danger if she stays there, or if medical help will not arrive at the scene of the accident.
- Raising the injured person's legs provides no benefit and can cause pain and further injury. **weak**
- Keep the person still.

Transportation

- If you have to move the injured person, hold her neck absolutely still. Do not twist the injure person's head, neck or body if you can avoid it. This is very important.
- If you have to move the injured person, put a folded blanket or clothing under the injured person when laying her back down to make her more comfortable. **weak**

Evidence that forms the basis for the recommendations

Injury to head, neck, or back

Evidence

- Neck pain together with age above 65 years, numbness or a tingling feeling in the extremities, or dangerous mechanism of injury are useful to indicate cervical trauma[(123), A].
- The effect of spinal immobilisation on mortality and injury in trauma victims is uncertain[124].
- Laying an injured person immobile on a hard surface is uncomfortable and makes conscious persons move more[(125), D].

Additional information

- Persons with potential head or spinal injury will often be brought to the hospital by basic first responders[28;126]. Teaching techniques on how to transport such victims is therefore important.

Broken or dislocated limbs

The response of an injured person to a broken bone can be very different. Some persons with a broken leg may still be able to walk with some pain. Another person may be unable to move. In some cases the broken or dislocated limb is an odd shape. If you are not sure, then it is safer to assume that a bone is broken or dislocated. Seek medical help.

What do you do?

1. Ask a bystander to seek help or to arrange for bringing the injured person to a medical care provider. Tell him to come back to you to confirm if help has been secured. The injured person urgently needs help. Shout or call for help if you are alone but do not leave the injured person.
2. If the break is bleeding seriously, press down on the bleeding or put on a compression bandage (see chapter 'Severe bleeding' p. 30).
3. If you suspect a broken leg, tell the injured person not to stand on it. Do not bandage the casualty if an ambulance is on its way.
4. If you suspect a broken arm, tell the injured person to immobilise his own arm by holding it close to his body until he obtains medical care. If the casualty cannot support an injured arm, turn up the lower end of the clothing and pin it above the arm to form a sling.
5. If the casualty has a broken leg and needs to be transported, keep the leg still by splinting it to the other leg:
   - carefully move the uninjured leg close to the injured leg;
   - use padding to fill in any hollow areas;
   - use bandaging or strips of cloth to attach the legs together.

   If both legs are broken: you need to use a splint, for example a stick.
6. If you use a splint, check that it is not too tight. If the fingers or toes become cold, white or blue, loosen the splint.
7. Keep the injured person warm, but do not overheat him. **weak**
8. Arrange urgent transport to medical care yourself if you are alone.
9. Once actions to obtain help have been made, stay with the injured person until medical help is available.
10. Keep checking that the injured person is conscious and breathing properly.

Caution

- If a limb looks odd or dislocated, do not try to reset it. This may make the injury worse.
- Raising the injured person's leg provides no benefit and can cause pain and further injury. Keep the person still. **weak**
- It is important to seek medical care straight away, as treatment by bonesetters sometimes can go very wrong, and an injured person can lose his limb or can die of complications.

When to seek medical help

If you are in doubt as to the severity of the injury, assume that the limb is broken or dislocated and seek medical help. **strong**

Evidence that forms the basis for the recommendations

Broken or dislocated limbs

Evidence

Injured persons or basic first responders are unable to decide themselves whether a bone is broken[1], and in particular to evaluate the ability to bear weight immediately after the injury[(127), C].

Additional information

- Informal care providers and bonesetters are regularly consulted for injured limbs[36;128]. Management by bonesetters typically includes the use of a bamboo stick splint and the topical application of herbs[110]. Many fractures heal properly, but complications, including gangrene, tetanus, non-union, malunion, can be very serious.

Injury to muscles or joints

Sudden unexpected movements like a blow or a fall can cause injuries to muscles or joints.

What do you do?

- Use ice, if you have it, to cool the injury. Ice can reduce pain and improve the healing.
- Wrap the ice in a cloth or a towel so it does not touch the skin directly.
- If you do not have ice, use cold water.
- Do not cool for more than 20 minutes at a time. **weak**

Caution

- Do not massage the injury.
- Do not put heat on the injury.
- Do not let the injured person continue his activity.
- Avoid alcohol.

When to seek medical help

Many injuries to muscles or joints can be managed at home. But if you are not sure how severe the injury is, it is safer to seek medical help.

Seek medical help in case of:

- bad bruising and swelling;
- some loss of feeling;
- inability to move the limb;
- a very painful and tender joint swelling straight after injury.

Seek medical care in the days after if:

- The injured person has difficulty walking or making other movements.
- The pain or swelling gets worse.
- The injured person has a fever and a swollen joint that feels warm to the touch;
- The injured person does not improve.

Evidence that forms the basis for the recommendations

Injury to muscles or joints

Evidence

- The evidence is inconclusive on the effectiveness of immediate post-injury cooling[(1)].
- In case of mild or moderate ankle sprains, elastic bandages have no significantly better outcomes than no bandages, and might even lead to worse outcomes[((129-132)), C)].
- In case of acute muscle injury, elastic bandages have no significantly better outcomes than minimal or no bandage[((133)), C)].

Additional information

- Experts consider that immediately after the accident, avoiding standing on an injured lower limb, or self-immobilisation of an injured upper limb, are often adequate and less painful than putting the limb in a bandage or a sling[(1)].

Eye injury

The eye is a person's window to the world. Eye injuries should always be managed with great care.

What do you do?

For *an object* that is stuck in the injured person's eye:

1. Ask a bystander to seek help or to arrange for bringing the injured person to a medical care provider. Tell him to come back to you to confirm if help has been secured. The injured person urgently needs help. Shout or call for help if you are alone but do not leave the injured person.
2. Do not try to remove it but loosely cover the eye.
3. Keep the head as still as possible.
4. Cover the good eye as well and ask the injured person to keep it still, because both eyes move together.
5. Arrange urgent transport to medical care yourself if you are alone.

For *a speck* in the injured person's eye:

1. If there is a speck in the eye, get the injured person to sit.
2. Rinse the speck out of the eye from the nose outwards.
3. Use clean water or water that has been boiled and cooled. Water at room temperature is more comfortable than cold water. Warm water might burn the eye.
4. If this does not work, cover the eye and seek medical assistance.

For *a blow* to the eye:

1. Make a cold compress:
   - put ice in a plastic bag and wrap it in a cloth
     or
   - soak a piece of clean cloth in cold water and squeeze it out.
   - make sure the eye is fully closed when applying the cold cloth.
2. Put this cold compress loosely on the eye as long as the person can tolerate it. Do not press hard against the eye. This helps with the pain and keeps down swelling.

For *harmful liquids* in the eye:

1. Ask a bystander to seek help or to arrange for bringing the injured person to a medical care provider. Tell him to come back to you to confirm if help has been secured. The injured person urgently needs help. Shout or call for help if you are alone but do not leave the injured person.
2. Rinse out the eye straight away with preferably clean water. Do this for 10 - 15 minutes, from the nose outwards so that none of the liquid runs out from one eye into the other.
3. Arrange urgent transport to medical care yourself if you are alone.

When to seek medical help

Eye injuries should always be managed with great care. Immediate medical help is needed for eye injuries if:

- a harmful liquid entered the eye;
- a speck cannot be removed or if an object (even very small) is stuck in the eye;
- the vision is affected.

If the condition of the eye gets worse in the days after, the injured person should seek medical help straight away. Signs to look for are:

- pain getting worse;
- vision that is getting worse;
- condition of the eye not getting better after 3 days.

Evidence that forms the basis for the recommendations

Eye injury

Evidence

- Warm fluids (32.2 °C to 37.8 °C) are more comfortable than fluids at room temperature (21°C) for irrigating the eye[(134), B]. The use of warm water may burn the eye and is therefore not recommended by the panel.
- Patching the eye following simple corneal abrasions does not improve healing or pain. In addition, the use of patches reduces the visual field.[(135), B]

Additional information

- People with eye injuries often delay seeking help. The main reason for delay was the consultation of non-specialised medical care or informal care first[33].

Bite wounds

Any human or animal bite (e.g. dog, cat, monkey) that breaks the skin needs special care because it can cause infections.

What do you do?

1. Wash your hands before giving first aid. Use soap to wash your hands or alternatively you can also use ash. Use soap to wash your hands or alternatively you can also use ash. Put on rubber gloves if available. You can also use a clean plastic bag. **strong**
2. If the person is bleeding severely, try to stop the bleeding first (see chapter 'Severe Bleeding' p.30).
3. Rinse the wound under running, clean water until it is clean.
4. Cover the wound with sterile gauze or a clean dry cloth.
5. Wash your hands after giving first aid. Use soap to wash your hands or alternatively you can also use ash. **strong**

When to seek medical help

Always seek medical help straight away for bite wounds because a person who has been bitten needs adequate medication to prevent infection. It is also important to be vaccinated for tetanus.

Evidence that forms the basis for the recommendations

Bite wounds

Evidence

- Antibiotics may prevent infections after human or animal bite wounds[(136;137), B].

Additional information

- There is no clear evidence on the actual risk of acquiring blood-borne viral infections from human bites[138] but experts recommend seeking immediate advice regarding post-exposure prophylaxis if the biter is likely to have a blood-borne viral infection[136].
- People are at risk of acquiring viral infections from animals if they have sustained bites or if they have had licks to broken skin[136].

Nose bleed

The nose contains small blood vessels that bleed easily. Therefore, nosebleeds are common. Either by a blow to the nose, or as a result of sneezing or picking the nose. But they can also occur without any apparent cause.

What do you do?

1. Ask the injured person to pinch his nose with index finger and thumb. Tell the injured person to breathe through the mouth.
   If necessary, pinch the injured person's nose yourself. Try not to touch the injured person's blood. Use gloves or a plastic bag.
2. Pinch for 10-15 minutes.
3. Ask the injured person to lean forwards so that he does not swallow or breathe in blood. Swallowing blood can make the person feel sick.
4. Wash your hands after giving first aid. Use soap to wash your hands or alternatively you can also use ash.

strong

When to seek medical help

In a few cases a nose bleed can be serious and lead to death. You should seek medical help if:

- blood is still coming from the nose after 20 minutes;
- the nose bleed was caused by a hard punch on the nose; a fall; a road accident;
- blood spurts from the nose:
- the injured person becomes sleepy or falls unconscious.

Evidence that forms the basis for the recommendations

Nose bleed

Evidence

- Ice on the back of the neck may reduce nasal blood flow[(139;140), D]. However the evidence is very limited and the panel does not recommend it in practice.

Additional information

- The anterior part of the nasal septum is the most common location of nosebleeds[141].
- Holding the head forward prevents the injured person from swallowing blood, which may cause nausea and vomiting[142].

Cuts and grazes

Even if an injured person just has a small cut or graze you still need to take care that the wound does not become infected.

What do you do?

1. Wash your hands before giving first aid. Use soap to wash your hands or alternatively you can also use ash. Put on rubber gloves if available. You can also use a clean plastic bag. **strong**
2. Try to stop or slow down the bleeding: press on the wound with a clean cloth or bandage.
3. Rinse out the wound with clean water. You can also use boiled and cooled water. **strong**
   ! Do not try to clean inside the wound by rubbing it. This may cause further damage to the wound.
4. Pour water on the wound until you cannot see any foreign material left in the wound. Foreign material means dirt or anything else that comes from outside the injured person's body.
5. If you have a piece of sterile gauze, then cover the wound with it.
6. Use a sterile plaster to close a clean cut.
   If no plaster is available, use a bandage or a clean cloth.
7. Bandage the dressing to the wound.Do not apply the bandage too firmly. If the part of the body below the bandage changes colour or is swelling, loosen the bandage a little bit.
8. Wash your hands after giving first aid. Use soap to wash your hands or alternatively you can also use ash. **strong**
9. Tell the injured person or the person caring for him to keep the wound dry. Do not allow flies to touch the wound. Keeping the wound clean will help, as a bad smell attracts flies.
10. Every 2 or 3 days rinse out the wound with clean water and change the dressing. If the wound is infected then clean this every day.

Caution

- Even small wounds need attention to prevent infection.
- If a dressing needs to be changed, do not tear the old one off as this can damage the healing wound.
- Instead, put enough water on the old dressing to take it off easily.
- It is not good to try to close a dirty wound.

When to seek medical help

Most cuts and grazes can be easily managed at home. You should seek medical help if:

- you cannot stop the bleeding;
- an object is in the wound;
- the wound has an irregular shape, is gaping open or is bigger than half the width of the injured person's hand;
- the injured person is losing feeling or has problems moving the body part;
- the wound is on the face, is on or near eyes, or in the area of the sex organs;
- the wound has dirt in it and cannot be cleaned properly;
- the wound has faeces or urine in it;
- the wound was caused by a bite;
- the injured person has diabetes or an immune disease;
- the injured person is 65 years old or older;
- it is more than 10 years since the injured person last had a tetanus injection or if there is any doubt about when the injured person last had a tetanus injection. Even small wounds can cause tetanus and it is very safe to get a tetanus injection.

It is best that wounds are managed within 6 hours. Do not delay seeking medical help.

Tell people *to watch out for infection* in the days after and get medical help if there is any sign of infection, such as:

- the pain is getting worse;
- the injured person has a fever or feels unwell;
- swollen, hot, red skin around the wound.

It is normal to have some light wound response. Get medical help if the seepage increases or is associated with signs of infection.

Evidence that forms the basis for the recommendations

Cuts and grazes

Evidence

- Irrigating wounds with drinkable water is effective to clean wounds[(143), B]. Boiled and cooled water can be used in the absence of potable tap water[(143), C].
- The evidence does not support routine administration of prophylactic antibiotics to prevent infection[(144), C].
- Adhesive strips are useful to close short simple wounds with minimal flexing, tension, or wetting[(145), B].
- The risk of infection is increased for elderly or persons with diabetes or immune disease; for wounds contaminated with dirt or an object; for wounds with an irregular shape, or with an increasing depth, length, and width; for bite wounds; as time from injury to repair increases[(145), C].

Additional information

- Application of antiseptics and antibacterials on wound tissue may be harmful[(1)].
- Harmful remedies include topical application of cow dung, dirt or soil. Their use as a cultural remedy must be discouraged[(46)].
- The risk of infection is increased in African countries and even small wounds need attention to prevent infection.
- Sterilised banana leaves might be an alternative means of dressing wounds[(119), C].
- Tetanus immunisation is needed for all wounds but fake or expired tetanus toxoids without potency are possibly being sold in pharmacies[(110)].

Bee or wasp stings

A bee or wasp sting causes a red, swollen lump that can be painful and itchy. Some people can have an allergic reaction which can be life-threatening.

What do you do?

1. Removing the sting as quick as possible can help keep the bite smaller. **weak**
2. Use your fingers or whatever you have at hand to remove the sting. **weak**
3. Wash the bite with water.
4. Use ice, if you have it, to cool the bite:
   - Wrap the ice in a cloth or a towel so it does not touch the skin directly.
   - If you do not have ice, use cold water.
   - Do not cool for more than 20 minutes at a time.

Caution

Do not scratch the bite: this can cause infections, especially when the nails are dirty.

When to seek medical help

Some people are allergic to bee or wasp stings. This is a life threatening situation. Seek emergency medical help if the injured person suffers the following symptoms right after being stung:

- a rapid pulse, dizziness or feeling faint;
- swelling or itching anywhere else on the body;
- difficulty breathing;
- a headache;
- difficulty swallowing, or swelling to the face or mouth.
- vomiting.

Seek medical attention if the sting is very painful.

Evidence that forms the basis for the recommendations

Bee or wasp stings

Evidence

- Quickly removing the sting may reduce the size of the weal[(146), D]. Pinching or scraping the sting away might not affect the size of the weal. A risk of scraping the sting is that it can break[(146), D].
- Topical aspirin in the treatment of bee and wasp stings appears to have no benefit and may increase the duration of redness[(147), C].
- We did not find evidence on the use of topical antihistamines to treat bee or wasp stings[(148)].

Poisoning

Poisoning can occur through several routes. Poisoning from swallowing is often caused by household products, overdose of medication or toxic plants. The effects of poisoning depend on what poison has been swallowed.

Signs of poisoning that can occur, include:
- nausea, vomiting, pain;
- difficulty breathing, seizures, confusion;
- odd skin colour.

What do you do?

1. Ask a bystander to seek help or to arrange for bringing the injured person to a medical care provider. Tell him to come back to you to confirm if help has been secured. The injured person urgently needs help. Shout or call for help if you are alone but do not leave the injured person.
2. Place the person on his left side. This will reduce absorption of the poison in the body. **weak**
3. Find out what the poisoned person has swallowed and when it happened.
4. Arrange urgent transport to medical care yourself if you are alone.
5. If this is safe, show the container of the poison to the doctor or write down the label.

Caution

- Avoid contact with any poisonous material on yourself.
- Do not force a poisoned person to vomit unless a nurse or doctor has told you to.
- Do not give milk or water to a poisoned person unless a nurse or doctor has told you to. This only helps for some poisons, and may cause harm in other cases.

Evidence that forms the basis for the recommendations

Poisoning

Evidence
- One study found that lying on the left side resulted in a significantly lower absorption compared to lying on the right side or sitting[(149), D].

Additional information
- Experts recommend not using charcoal tablets from pharmacies and only forcing a poisoned person to vomit or to drink milk under medical advice[150].

Emergency childbirth

In the last part of pregnancy a woman can go in labour at any time.
The following signs mean that labour has started and that the baby is coming:

- *painful contractions and any of the following;*
- *the waters break;*
- *sticky discharge;*
- *abdominal discomfort;*
- *local back pains.*

Labour usually is a lengthy process so in many cases there is time to get the mother to medical aid before the baby arrives.

What do you do?

What do you do when *labour starts*?

1. If there is medical care in your area, it is best to arrange for bringing the woman there.If not, send someone for the help of a skilled attendant and prepare for the delivery. If available to you, take your delivery kit. This kit contains soap, gloves, materials for cutting the cord and other objects to guarantee a clean delivery and to prevent infections of the baby after birth.
2. Offer support and personal care: **strong**
   - Encourage the woman's birth companion(s) to be involved.
   - Praise and encourage her.
   - Protect and respect her privacy.
   - Massaging the woman's back may help with the pain.
3. Encourage the woman to move around and find the most comfortable positions.
4. Encourage the woman to urinate often. This will help make more space for the baby.
5. Encourage the woman to drink during labour, even at the end. She can also eat a little to keep up her strength. **weak**
6. Encourage her to think about her breathing:
   - To breathe out slowly and loudly, and to relax with each breath.
   - Suggest breathing more slowly if the woman feels dizzy, unwell, or tingling in her face, hands or feet. **weak**
7. Seek medical help immediately:
   - if the baby presents with the buttocks or feet first, instead of the head first;
   - if there are no contractions 6 hours after the waters broke;
   - if contractions continue for more than 12 hours;
   - the woman is bleeding or has a fever.

What do you do when *the pushing stage* comes?

1. Help the woman into the most comfortable position. An upright position is best, but a lying position is the least challenging to assist with the delivery. If the woman lies on her back, it is best to put a small pillow under the right hip so that she lies to the side. The weight of the baby may press on important blood vessels. **weak**
2. Wash your hands before giving first aid. Use soap to wash your hands or alternatively you can also use ash. Put on rubber gloves if available. You can also use a clean plastic bag. **strong**
3. Women naturally feel the urge to push. If pushing is not working then the woman should change position or empty her bladder. Ask the woman not to push when the baby's head is being delivered.
4. Watch the baby come out while supporting the head and shoulders.

   ! Do not do anything to 'pull' the baby out.

Caution

- Do not leave the woman alone.
- Do not use any remedies or medications to speed up labour or to clear out the bowel unless a midwife or doctor has told you to.
- Do not push on the woman's belly during labour or after delivery.

What do you do?

What do you do when *the baby is born*?

1. Immediately place him on the mother's bare chest or abdomen so they have skin-to-skin contact. **strong**
2. Cut the baby's cord:
   - Use 2 long ties and a clean knife, scissors or razor blade. The material you use to cut the cord should be sterilised by placing it 10 minutes in boiling water or running it through a flame a few times.
   - Tie the first knot 2 cm (2 fingers) away from the child's abdomen.
   - Tie the second knot 5 cm (5 fingers) away from the abdomen.
   - Add another tie on the side of the baby, if the cord continues bleeding after cutting it.
   - Keep the cord dry and clean. **strong**

   ! Do not put any substance on the baby's cord or stump. This could lead to infection.
3. Wipe the baby clean and dry him.
4. The mother and baby must be kept warm and close together. The baby can be dressed or wrapped.
5. The placenta or afterbirth will come on its own. When it is delivered, put it somewhere safe until it can be disposed of appropriately.

   ! Do not try to pull out the afterbirth by pulling on the cord. If the placenta rips, it may cause infection.
6. Wash your hands after giving first aid. Use soap to wash your hands or alternatively you can also use ash. **strong**
7. Encourage breast-feeding immediately after birth. Although there is no breast milk, the baby's sucking will encourage the milk to come. Breast-feeding helps the placenta to come out and prevents bleeding of the woman after delivery. **strong**
8. Encourage the woman to move around as soon as she feels able after the birth.
9. Do not leave the mother alone during the first 24 hours. The woman should always seek medical attention.

What do you do if *a baby is not breathing*?

1. If the baby is not breathing or is struggling to breathe, start resuscitation within 1 minute of birth.
2. Move the baby to a clean, dry and warm surface.
3. Tell the mother that the baby is having problems breathing and that you will help him to breathe.
4. Keep the baby wrapped up warm.
5. Start resuscitation as described in first aid for breathing arrest (see chapter 'No breathing' p. 21).
6. Stop resuscitation after 20 minutes if the baby is not breathing or gasping for air. Explain what has happened to the mother and offer your support.

What do you do if *the woman is bleeding* heavily after delivery?

1. Seek immediate medical help.
2. Massage the belly firmly below the navel.
3. Ask the woman to urinate, if possible.

These actions can help to slow down the bleeding.

When to seek medical help

Even though giving birth is a natural part of life, it can sometimes threaten the life of the mother or baby.

When to seek medical help for *the mother*?

The mother should always seek medical attention after delivery.

Go to the hospital or health centre *WITHOUT DELAY*, day or night, if the mother has any of the following danger signs:

- fever and weakness, unable to get out of bed;
- pain in the belly and/or bad smelling substance from the vagina;
- sudden bleeding or increasing loss of blood;
- fits;
- difficulty breathing, fast breathing or chest pain;
- the mother can feel her heart beating too hard or irregularly;
- terrible headaches and blurry sight;
- nausea, vomiting;
- faintness, dizziness.
- if the afterbirth is incomplete or has not been delivered 1 hour after the birth of the baby.

When to seek medical help for *the baby*?

A newborn baby should always get medical attention.

Seek medical help *WITHOUT DELAY*, day or night, if the baby:

- is very small;
- has difficulty breathing;
- has fits (see chapter 'Fits' p. 26);
- has fever (see chapter 'Fever' p. 23);
- feels cold;
- is bleeding from cord stump;
- is not able to breast-feed.

Transportation

If you must travel with the baby to get medical help:

- keep him warm against the skin of his mother or someone else;
- keep him covered with a blanket and his head covered with a cap;
- protect the baby from direct sunlight;
- encourage the mother to breast-feed during the journey. If the baby cannot breast-feed and the journey is longer than 3 hours, get the mother to squeeze breast milk into a cup and feed the baby by cup.

Evidence that forms the basis for the recommendations

Emergency childbirth

WHAT DO YOU DO WHEN LABOUR STARTS?

Evidence

- There is a lack of evidence to suggest that the mother's position or mobility during the first stage of labour affects outcomes[(151)].
- There is evidence that remaining supine in the second stage of labour negatively affects outcomes for the mother and baby. There is also evidence that the hands-and-knees position helps relieve pain for the mother in the second stage of labour and has no adverse effects[((151), B)]. The guideline panel held the view that the supine position is the least challenging for basic first responders assisting in emergency childbirth.
- Women that eat during labour vomit more often, but there are no significant differences in outcomes for the mother and baby[((151), B)].
- There is no evidence that breathing and relaxation techniques reduce labour pain or affect the labour experience[((151), C)]. There is limited evidence that massage and reassuring touch reduces pain and anxiety during labour[((151), B)].
- Application of warm compresses during labour does not appear to improve perineal outcomes[((151), C)].

Additional information

- Medicinal plants are sometimes used to induce labour. Using the wrong dosage can lead to poisoning and may threaten the life of the unborn baby and the mother[(152)].

WHAT DO YOU DO WHEN THE BABY IS BORN?

Evidence

- Delaying cord clamping for at least three minutes reduces the risk of anaemia in babies[((151;153;154), A)] and does not seem to influence the occurrence of post partum haemorrhage in mothers[((153;154), B)]. However, the panel does not consider that this is feasible within the context of basic first responders.
- Use of antiseptics may reduce concern of mothers about the cord, but in comparison with keeping the cord dry and clean it does not reduce the risk of infection.[((73), C)]
- Early skin-to-skin contact appears to improve breast-feeding outcomes and crying of babies[((73), B)].
- It appears that babies who are initiated to breast-feeding within one day of birth are significantly less likely to die in the neonatal period compared with those who are initiated after one day[((155), C)].
- It appears that initiation of breast-feeding as soon as possible after birth protects newborn babies from low body temperatures.[((156), B)]

Additional information

- Delayed breast-feeding may result from the idea that there is not enough milk, the baby should be washed first, the baby is not hungry or the baby should sleep[(157)].

WHAT DO YOU DO IF A BABY IS NOT BREATHING?

- The recommendations for resuscitation of newborns are extracted from the ILCOR guidelines[(16)].

WHAT DO YOU DO IF THE WOMAN IS BLEEDING HEAVILY AFTER DELIVERY?

Evidence

- There is no evidence for the effectiveness of immediate suckling to prevent postpartum blood loss[((158), C)].
- Stimulating the nipples immediately after delivery might reduce the incidence of postpartum haemorrhage[((159), C)].

Additional information

- A full bladder may prevent contraction of the uterus and can increase bleeding[(160)].

Acknowledgements and references

Acknowledgements

Expert panel: Volmink Jimmy (chair) (South African Cochrane Centre, Stellenbosch University, Medical Research Council, South Africa), Agbro Benson (Nigerian Red Cross), Ahmed Omar (American International Health Alliance - Twinning Centre, Ethiopia), Bakker Jenny (South African Red Cross), Bitex Okot Paul (Uganda Red Cross), Gichuhi Stephen (University of Nairobi, Kenya), Jaoko Walter (University of Nairobi, Kenya), Khamati Anekha Sylvia (Kenya Red Cross), Macgregor Hayley (University of Sussex, United Kingdom), Naidoo Navin (Cape Peninsula University of Technology, South Africa), Sebaganwa Jérôme (Rwandan Red Cross), Vandekerckhove Philippe

Project management: De Buck Emmy, Vandekerckhove Philippe, Vande Veegaete Axel, Van de Velde Stijn, Verhelst Katja

Evidence reviewers: De Buck Emmy, Van de Velde Stijn

Text and editing: Commotie, De Buck Emmy, Van de Velde Stijn

Peer reviewers: Bottieau Emmanuel (Institute of Tropical Medicine Antwerp, Belgium) Cleymans An, De Weggheleire Anja (Institute of Tropical Medicine Antwerp, Belgium), De Winne Krista, Duysburgh Els (International Centre for Reproductive Health, Belgium), Meeremans Leen, Sermon An (University Hospitals Leuven, Belgium), Temmerman Marleen (International Centre for Reproductive Health, Belgium), Van Gaever Liesbet, Wallis Lee (Stellenbosch University, University of Cape Town, South Africa)

Photography: Bleuckx Linda, Croes Stephan, Neuenburg Grant Lee (Freelance photographer), Van Hooreweder Marc

Design and drawings: Commotie, Craen Roel, Vanhorenbeeck Geert

Other contributors: Boelaert Freya, Bryssinck Maaike, Goeminne Dries, Henderickx Anne, Mortier Dries (University Hospitals Leuven), Smallberger Carine (Stellenbosch Univeristy, South Africa), Struys Paul, Van de Velde Tony (No affiliation), Vangoethem Marc, Wouters Mireille, Young Taryn (RYTD Consultancy, South Africa)

Thanks to all the models that have taken time to contribute to this publication.

If no affiliation is mentioned, the person is affiliated to the Belgian Red Cross-Flanders.

References

(1) Van de Velde S, Broos P, Van Bouwelen M, De Win R, Sermon A, Verduyckt J, et al. European first aid guidelines. Resuscitation 2007;72(2):240-51.

(2) Laxminarayan R, Mills AJ, Breman JG, Measham AR, Alleyne G, Claeson M, et al. Advancement of global health: key messages from the Disease Control Priorities Project. Lancet 2006;367(9517):1193-208.

(3) Tiska MA, du-Ampofo M, Boakye G, Tuuli L, Mock CN. A model of prehospital trauma training for lay persons devised in Africa. Emerg Med J 2004;21(2):237-9.

(4) Mock CN, Tiska M, du-Ampofo M, Boakye G. Improvements in prehospital trauma care in an African country with no formal emergency medical services. J Trauma 2002;53(1):90-7.

(5) Richards T. Poor countries lack relevant health information, says Cochrane editor. BMJ 2004;328(7435):310-b.

(6) Richards T, Tumwine J. Poor countries make the best teachers: discuss. BMJ 2004;329(7475):1113-4.

(7) Scottish Intercollegiate Guidelines Network. SIGN 50: A guideline developer's handbook. Edinburgh: SIGN. 2008. Available from: www.sign.ac.uk/guidelines.

(8) Raine R, Sanderson C, Black N. Developing clinical guidelines: a challenge to current methods. BMJ 2005;331(7517):631-3.

(9) Disease control priorities in developing counries. Second edition ed. Copublication of Oxford University Press and The World Bank; 2006.

(10) Global burden of disease and risk factors. New York:Oxford University Press.; 2006.

(11) Bertrand I, Hunter L. African Index Medicus--a cooperative undertaking. Health Libr Rev 1998;15(1):17-20.

(12) Siegfried N, Clarke M, Volmink J. Randomised controlled trials in Africa of HIV and AIDS: descriptive study and spatial distribution. BMJ 2005;331(7519):742.

(13) The data collection checklist. Cochrane Effective Practice and Organisation of Care Group. 2002. Available from: http://www.epoc.cochrane.org/files/website%20files/Documents/Reviewer%20 Resources/datacollectionchecklist.pdf.

(14) Formulier voor het beoordelen van een cohortonderzoek. Dutch Cochrane Centre. 2010. Available from: www.cochrane.nl.

(15) Whiting P, Rutjes AW, Reitsma JB, Bossuyt PM, Kleijnen J. The development of QUADAS: a tool for the quality assessment of studies of diagnostic accuracy included in systematic reviews. BMC Med Res Methodol 2003;3:25.

(16) Koster RW, Sayre MR, Botha M, Cave DM, Cudnik MT, Handley AJ, et al. Part 5: Adult basic life sup port: 2010 International consensus on cardiopulmonary resuscitation and emergency cardiovascular care science with treatment recommendations.Resuscitation. 2010 Oct;81 Suppl 1:e48-70.

(17) Grading quality of evidence and strength of recommendations. BMJ 2004;328(7454):1490.

(18) Guyatt GH, Oxman AD, Vist GE, Kunz R, Falck-Ytter Y, onso-Coello P, et al. GRADE: an emerging consensus on rating quality of evidence and strength of recommendations. BMJ 2008;336(7650):924-6.

(19) Sabuni LP. Dilemma with the local perception of causes of illnesses in central Africa: muted concept but prevalent in everyday life. Qual Health Res 2007;17(9):1280-91.

(20) Kelly JM, Osamba B, Garg RM, Hamel MJ, Lewis JJ, Rowe SY, et al. Community health worker performance in the management of multiple childhood illnesses: Siaya District, Kenya, 1997-2001. Am J Public Health 2001;91(10):1617-24.

(21) Mshana G, Hampshire K, Panter-Brick C, Walker R. Urban-rural contrasts in explanatory models and treatment-seeking behaviours for stroke in Tanzania. J Biosoc Sci 2008;40(1):35-52.

(22) Ellis AA, Winch P, Daou Z, Gilroy KE, Swedberg E. Home management of childhood diarrhoea in southern Mali-Implications for the introduction of zinc treatment. Social Science and Medicine 2007;64(3):701-12.

(23) Beiersmann C, Sanou A, Wladarsch E, De AM, Kouyate B, Muller O. Malaria in rural Burkina Faso: local illness concepts, patterns of traditional treatment and influence on health-seeking behaviour. Malar J 2007;6:106.

(24) Hundt GL, Stuttaford M, Ngoma B. The social diagnostics of stroke-like symptoms: healers, doctors and prophets in Agincourt, Limpopo Province, South Africa. J Biosoc Sci 2004;36(4):433-43.

(25) Pilkington H, Mayombo J, Aubouy N, Deloron P. Malaria, from natural to supernatural: a qualitative study of mothers' reactions to fever (Dienga, Gabon). J Epidemiol Community Health 2004;58(10):826-30.

(26) Montgomery CM, Mwengee W, Kong'ong'o M, Pool R. 'To help them is to educate them': power and pedagogy in the prevention and treatment of malaria in Tanzania. Trop Med Int Health 2006;11(11):1661-9.

(27) Spahn DR, Cerny V, Coats TJ, Duranteau J, Fernandez-Mondejar E, Gordini G, et al. Management of bleeding following major trauma: a European guideline. Crit Care 2007;11(1):R17.
(28) Saidi HS. Initial injury care in Nairobi, Kenya: a call for trauma care regionalisation. East Afr Med J 2003;80(9):480-3.
(29) Awad A, Eltayeb I, Matowe L, Thalib L. Self-medication with antibiotics and antimalarials in the community of Khartoum State, Sudan. J Pharm Pharm Sci 2005;8(2):326-31.
(30) Kallander K, Hildenwall H, Waiswa P, Galiwango E, Peterson S, Pariyo G. Delayed care seeking for fatal pneumonia in children aged under five years in Uganda: a case-series study. Bull World Health Organ 2008;86(5):332-8.
(31) Diallo D, Graz B, Falquet J, Traore AK, Giani S, Mounkoro PP, et al. Malaria treatment in remote areas of Mali: use of modern and traditional medicines, patient outcome. Trans R Soc Trop Med Hyg 2006;100(6):515-20.
(32) Bertrand E, Muna WFT, Diouf SM, Ekra A, Kane A, Kingue S, et al. Cardiovascular emergencies in Subsaharan Africa. Arch Mal Coeur Vaiss 2006;99(12):1159-65.
(33) Mensah A, Fany A, Adjorlolo C, Toure ML, Kasieu GM, Mihluedo KA, et al. Epidemiology of eye injuries in Abidjanian children. Sante 2004;14(4):239-43.
(34) Sloan DJ, Dedicoat MJ, Lalloo DG. Healthcare-seeking behaviour and use of traditional healers after snakebite in Hlabisa sub-district, KwaZulu Natal. Trop Med Int Health 2007;12(11):1386-90.
(35) Bazzano AN, Kirkwood BR, Tawiah-Agyemang C, Owusu-Agyei S, Adongo PB. Beyond symptom recognition: care-seeking for ill newborns in rural Ghana. Trop Med Int Health 2008;13(1):123-8.
(36) Aries MJ, Joosten H, Wegdam HH, van der GS. Fracture treatment by bonesetters in central Ghana: patients explain their choices and experiences. Trop Med Int Health 2007;12(4):564-74.
(37) Spangenberg K, Mock C. Utilization of health services by the injured residents in Kumasi, Ghana. Int J Inj Contr Saf Promot 2006;13(3):194-6.
(38) Scaling up home-based management of malaria: from research to implementation. World Health Organization. 2004. Available from: http://apps.who.int/tdr/publications/training-guideline-publications/scaling-up-home-based-management/pdf/home_2004.pdf .
(39) Krasovec K. Auxiliary technologies related to transport and communication for obstetric emergencies. Int J Gynaecol Obstet 2004;85 Suppl 1:S14-S23.
(40) Mrisho M, Schellenberg JA, Mushi AK, Obrist B, Mshinda H, Tanner M, et al. Factors affecting home delivery in rural Tanzania. Trop Med Int Health 2007;12(7):862-72.
(41) Winch PJ, Gilroy KE, Doumbia S, Patterson AE, Daou Z, Diawara A, et al. Operational issues and trends associated with the pilot introduction of zinc for childhood diarrhoea in Bougouni district, Mali. J Health Popul Nutr 2008;26(2):151-62.
(42) Rabie T, Curtis V. Handwashing and risk of respiratory infections: a quantitative systematic review. Tropical Medicine and International Health 2006;11(3):258-67.
(43) Ejemot RI, Ehiri JE, Meremikwu MM, Critchley JA. Hand washing for preventing diarrhoea. Cochrane Database Syst Rev 2008;(1):CD004265.
(44) Hoque BA, Briend A. A comparison of local handwashing agents in Bangladesh. J Trop Med Hyg 1991;94(1):61-4.
(45) Hoque BA, Mahalanabis D, Alam MJ, Islam MS. Post-defecation handwashing in Bangladesh: practice and efficiency perspectives. Public Health 1995;109(1):15-24.
(46) Albertyn R, Bickler SW, Rode H. Paediatric burn injuries in Sub Saharan Africa--an overview. Burns 2006;32(5):605-12.
(47) Kober A, Scheck T, Schubert B, Strasser H, Gustorff B, Bertalanffy P, et al. Auricular acupressure as a treatment for anxiety in prehospital transport settings. Anesthesiology 2003;98(6):1328-32.
(48) Scholes C, Turpin G, Mason S. A randomised controlled trial to assess the effectiveness of providing self-help information to people with symptoms of acute stress disorder following a traumatic injury. Behav Res Ther 2007;45(11):2527-36.
(49) Turpin G, Downs M, Mason S. Effectiveness of providing self-help information following acute traumatic injury: randomised controlled trial. Br J Psychiatry 2005;187:76-82.
(50) Bernardo LM, Henker R, O'Connor J. Treatment of trauma-associated hypothermia in children: evidence-based practice. Am J Crit Care 2000;9(4):227-34.
(51) Watts DD, Roche M, Tricarico R, Poole F, Brown JJ, Jr., Colson GB, et al. The utility of traditional prehospital interventions in maintaining thermostasis. Prehosp Emerg Care 1999;3(2):115-22.
(52) Brady M, Kinn S, O'Rourke K, Randhawa N, Stuart P. Preoperative fasting for preventing perioperative complications in children. Cochrane Database Syst Rev 2005;(2):CD005285.
(53) Brady M, Kinn S, Stuart P. Preoperative fasting for adults to prevent perioperative complications. Cochrane Database Syst Rev 2003;(4):CD004423.

(54) Stroke: National clinical guideline for diagnosis and initial management of acute stroke and transient ischaemic attack (TIA). The National Collaborating Centre for Chronic Conditions. 2008. Available from: http://www.nice.org.uk/nicemedia/pdf/CG68NICEGuideline.pdf.
(55) Baigent C, Blackwell L, Collins R, Emberson J, Godwin J, Peto R, et al. Aspirin in the primary and secondary prevention of vascular disease: collaborative meta-analysis of individual participant data from randomised trials. Lancet 2009;373(9678):1849-60.
(56) Bruyninckx R, Aertgeerts B, Bruyninckx P, Buntinx F. Signs and symptoms in diagnosing acute myocardial infarction and acute coronary syndrome: a diagnostic meta-analysis. British Journal of General Practice 2008;58(547):105-11.
(57) Feldman M, Cryer B. Aspirin absorption rates and platelet inhibition times with 325-mg buffered aspirin tablets (chewed or swallowed intact) and with buffered aspirin solution. Am J Cardiol 1999;84(4):404-9.
(58) Erhardt L, Herlitz J, Bossaert L, Halinen M, Keltai M, Koster R, et al. Task force on the management of chest pain. Eur Heart J 2002;23(15):1153-76.
(59) Sayre MR, Berg RA, Cave DM, Page RL, Potts J, White RD. Hands-only (compression-only) cardio-pulmonary resuscitation: a call to action for bystander response to adults who experience out-of-hospital sudden cardiac arrest: a science advisory for the public from the American Heart Association Emergency Cardiovascular Care Committee. Circulation 2008;117(16):2162-7.
(60) Position statement on recent CPR research findings. Resuscitation Council of Southern Africa. 2008. Available from: www.resuscitationcouncil.co.za/Position3.doc.
(61) Part 10: First aid. Circulation 2005;112(22 SUPPL.):III115-III125.
(62) Wood C, Foëx BA. Near drowning and antibiotics. BestBETs. 2005. Available from: http://www.bestbets.org/bets/bet.php?id=120.
(63) Teng CL, Ng CJ, Nik-Sherina H, Zailinawati AH, Tong SF. The accuracy of mother's touch to detect fever in children: a systematic review. J Trop Pediatr 2008;54(1):70-3.
(64) Katz-Sidlow RJ, Rowberry JP, Ho M. Fever determination in young infants: prevalence and accuracy of parental palpation. Pediatr Emerg Care 2009;25(1):12-4.
(65) Meremikwu M, Oyo-Ita A. Paracetamol for treating fever in children. Cochrane Database Syst Rev 2002;(2):CD003676.
(66) Meremikwu M, Oyo-Ita A. Physical methods for treating fever in children. Cochrane Database Syst Rev 2003;(2):CD004264.
(67) WHO Guidelines for the treatment of malaria. World Health Organization. 2006. Available from: http://whqlibdoc.who.int/publications/2010/9789241547925_eng.pdf.
(68) Simoes EA, McGrath EJ. Recognition of pneumonia by primary health care workers in Swaziland with a simple clinical algorithm. Lancet 1992;340(8834-8835):1502-3.
(69) Redd SC, Vreuls R, Metsing M, Mohobane PH, Patrick E, Moteetee M. Clinical signs of pneumonia in children attending a hospital outpatient department in Lesotho. Bull World Health Organ 1994;72(1):113-8.
(70) Weber MW, Mulholland EK, Jaffar S, Troedsson H, Gove S, Greenwood BM. Evaluation of an algorithm for the integrated management of childhood illness in an area with seasonal malaria in the Gambia. Bull World Health Organ 1997;75 Suppl 1:25-32.
(71) Chest infections. Sowerby Centre for Health Informatics at Newcastle. 2007. Available from: http://www.cks.nhs.uk/chest_infections_adult.
(72) Acute cough with chest signs in children. Sowerby Centre for Health Informatics at Newcastle. 2007. Available from: http://www.cks.nhs.uk/cough_acute_with_chest_signs_in_children/how_up_to_date_is_this_topic/knowledge_update/new_evidence.
(73) Demott K, Bick D, Norman R, Ritchie G, Turnbull N, Adams C, et al. Clinical guidelines and evidence review for post natal care: routine post natal care of recently delivered women and their babies. National Collaborating Centre For Primary Care And Royal College Of General Practitioners. 2006. Available from: http://www.nice.org.uk/nicemedia/pdf/CG037fullguideline.pdf.
(74) Makundi EA, Malebo HM, Mhame P, Kitua AY, Warsame M. Role of traditional healers in the management of severe malaria among children below five years of age: the case of Kilosa and Handeni Districts, Tanzania. Malar J 2006;5:58.
(75) Ibidapo CA. Perception of causes of malaria and treatment-seeking behaviour of nursing mothers in a rural community. Aust J Rural Health 2005;13(4):214-8.
(76) Hopkins H, Talisuna A, Whitty CJ, Staedke SG. Impact of home-based management of malaria on health outcomes in Africa: a systematic review of the evidence. Malar J 2007;6(1):134.
(77) Kidane G, Morrow RH. Teaching mothers to provide home treatment of malaria in Tigray, Ethiopia: a randomised trial. Lancet 2000;356(9229):550-5.
(78) Kouyate B, Some F, Jahn A, Coulibaly B, Eriksen J, Sauerborn R, et al. Process and effects of a community intervention on malaria in rural Burkina Faso: randomized controlled trial. Malar J 2008;7:50.

(79) Ajayi IO, Falade CO, Bamgboye EA, Oduola AM, Kale OO. Assessment of a treatment guideline to improve home management of malaria in children in rural south-west Nigeria. Malar J 2008;7:24.
(80) Hildenwall H, Rutebemberwa E, Nsabagasani X, Pariyo G, Tomson G, Peterson S. Local illness concepts--implications for management of childhood pneumonia in eastern Uganda. Acta Trop 2007;101(3):217-24.
(81) Kallander K, Tomson G, Nsabagasani X, Sabiiti JN, Pariyo G, Peterson S. Can community health workers and caretakers recognise pneumonia in children? Experiences from western Uganda. Trans R Soc Trop Med Hyg 2006;100(10):956-63.
(82) Kallander K, Nsungwa-Sabiiti J, Peterson S. Symptom overlap for malaria and pneumonia--policy implications for home management strategies. Acta Trop 2004;90(2):211-4.
(83) El-Radhi AS, Barry W. Do antipyretics prevent febrile convulsions? Arch Dis Child 2003;88(7):641-2.
(84) Meremikwu MM, Oyo IA. Paracetamol for treating fever in children. Cochrane Database Syst Rev 2002: (2):CD003676.
(85) Ndukwe KC, Folayan MO, Ugboko VI, Elusiyan JB, Laja OO. Orofacial injuries associated with prehospital management of febrile convulsion in Nigerian children. Dent Traumatol 2007;23(2):72-5.
(86) Mbewe E, Haworth A, Atadzhanov M, Chomba E, Birbeck GL. Epilepsy-related knowledge, attitudes, and practices among Zambian police officers. Epilepsy Behav 2007;10(3):456-62.
(87) Okeke TA, Okafor HU, Uzochukwu BS. Traditional healers in Nigeria: perception of cause, treatment and referral practices for severe malaria. J Biosoc Sci 2006;38(4):491-500.
(88) Millogo A, Ratsimbazafy V, Nubukpo P, Barro S, Zongo I, Preux PM. Epilepsy and traditional medicine in Bobo-Dioulasso (Burkina Faso). Acta Neurol Scand 2004;109(4):250-4.
(89) Moshi MJ, Kagashe GA, Mbwambo ZH. Plants used to treat epilepsy by Tanzanian traditional healers. J Ethnopharmacol 2005;97(2):327-36.
(90) Gastroenteritis. Sowerby Centre for Health Informatics at Newcastle. 2007. Available from: http://www.cks.nhs.uk/gastroenteritis.
(91) Bhatnagar S, Lodha R, Choudhury P, Sachdev HP, Shah N, Narayan S, et al. IAP Guidelines 2006 on management of acute diarrhea. Indian Pediatr 2007;44(5):380-9.
(92) Kenya PR, Muttunga JN, Mwenesi H, Molla AM, Bari A, Juma R, et al. Comparison of safety of glucose oral rehydration solution and maize oral rehydration therapy for home management of diarrhoea in Kenya. J Trop Pediatr 2001;47(4):226-9.
(93) Hahn S, Kim S, Garner P. Reduced osmolarity oral rehydration solution for treating dehydration caused by acute diarrhoea in children. Cochrane Database Syst Rev 2002;(1):CD002847.
(94) Pruvost I, Dubos F, Aurel M, Hue V, Martinot A. Value of history and clinical and laboratory data for the diagnosis of dehydration due to acute diarrhea in children younger than 5 years. Presse Med 2008;37(4 Pt 1):600-9.
(95) Accorsi S, Fabiani M, Ferrarese N, Iriso R, Lukwiya M, Declich S. The burden of traditional practices, ebino and tea-tea, on child health in Northern Uganda. Soc Sci Med 2003;57(11):2183-91.
(96) Kikwilu EN, Hiza JF. Tooth bud extraction and rubbing of herbs by traditional healers in Tanzania: prevalence, and sociological and environmental factors influencing the practices. Int J Paediatr Dent 1997;7(1):19-24.
(97) Shann F. Meta-analysis of trials of prophylactic antibiotics for children with measles: inadequate evidence. BMJ 1997;314:334-6.
(98) Lebese RT, Netshandama VO, Shai-Mahoko NS. Cultural health practices of South African Vatsonga people on the home care of children with measles. Curationis 2004;27(1):52-64.
(99) Kragh JF, Jr., Walters TJ, Baer DG, Fox CJ, Wade CE, Salinas J, et al. Practical use of emergency tourniquets to stop bleeding in major limb trauma. J Trauma 2008;64(2 Suppl):S38-S49.
(100) Kettaneh N, Jones JS. Use of the Trendelenburg position to improve hemodynamics during hypovolemic shock. BestBETs. 2008. Available from: http://www.bestbets.org/bets/bet.php?id=1710.
(101) Simpson ID, Tanwar PD, Andrade C, Kochar DK, Norris RL. The Ebbinghaus retention curve: training does not increase the ability to apply pressure immobilisation in simulated snake bite--implications for snake bite first aid in the developing world. Trans R Soc Trop Med Hyg 2008;102(5):451-9.
(102) Norris RL, Ngo J, Nolan K, Hooker G. Physicians and lay people are unable to apply pressure immobilization properly in a simulated snakebite scenario. Wilderness Environ Med 2005;16(1):16-21.
(103) Howarth DM, Southee AE, Whyte IM. Lymphatic flow rates and first-aid in simulated peripheral snake or spider envenomation. Med J Aust 1994;161(11-12):695-700.
(104) Anker RL, Straffon WG, Loiselle DS, Anker KM. Snakebite. Comparison of three methods designed to delay uptake of 'mock venom'. Aust Fam Physician 1983;12:365-8.
(105) Anker RL, Straffon WG, Loiselle DS, Anker KM. Retarding the uptake of "mock venom" in humans: comparison of three first-aid treatments. Med J Aust 1982;1(5):212-4.
(106) Pe T, Mya S, Myint AA, Aung NN, Kyu KA, Oo T. Field trial of efficacy of local compression immobilization first-aid technique in Russell's viper (Daboia russelii siamensis) bite patients. Southeast Asian J Trop Med Public Health 2000;31(2):346-8.

(107) Chippaux JP, Diedhiou I, Stock R. Study of the action of black stone (also known as snakestone or serpent stone) on experimental envenomation. Sante 2007;17(3):127-31.
(108) Drame B, Diani N, Togo MM, Maiga M, Diallo D, Traore A. Envenomation accidents caused by snake-bites in the surgical emergency unit of Gabriel-Toure Hospital, Bamako, Mali (1998-1999). Bull Soc Pathol Exot 2005;98(4):287-9.
(109) Ehui E, Kra O, Ouattara I, Tanon A, Kassi A, Eholie S, et al. Generalized tetanus complicating a traditional medicine applied for snakebite. Bull Soc Pathol Exot 2007;100(3):184-5.
(110) Udosen AM, Ugare UG, Ekpo R. Generalized tetanus complicating lower limb fractures managed by traditional bone healers. Trop Doct 2005;35(4):237-9.
(111) Burns and scalds. Sowerby Centre for Health Informatics at Newcastle. 2007. Available from: http://www.cks.nhs.uk/burns_and_scalds.
(112) Maenthaisong R, Chaiyakunapruk N, Niruntraporn S, Kongkaew C. The efficacy of aloe vera used for burn wound healing: a systematic review. Burns 2007;33(6):713-8.
(113) Jull AB, Rodgers A, Walker N. Honey as a topical treatment for wounds. Cochrane Database Syst Rev 2008; (4):CD005083.
(114) Ingle R, Levin J, Polinder K. Wound healing with honey: a randomised controlled trial. S Afr Med J 2006;96(9):831-5.
(115) Pieper B, Caliri MH. Nontraditional wound care: A review of the evidence for the use of sugar, papaya/papain, and fatty acids. J Wound Ostomy Continence Nurs 2003;30(4):175-83.
(116) Sheridan RL, Petras L, Lydon M, Salvo PM. Once-daily wound cleansing and dressing change: efficacy and cost. J Burn Care Rehabil 1997;18(2):139-40.
(117) De Souza BA, Furniss D, Olaofe G, Jawad M. Vaseline and burns: vaseline should not be used as first aid for burns. BMJ 2003;327(7426):1289.
(118) Ryan TJ. Wound healing in the developing world. Dermatol Clin 1993;11(4):791-800.
(119) Gore MA, Akolekar D. Evaluation of banana leaf dressing for partial thickness burn wounds. Burns 2003;29(5):487-92.
(120) Keswani MH, Vartak AM, Patil A, Davies JW. Histological and bacteriological studies of burn wounds treated with boiled potato peel dressings. Burns 1990;16(2):137-43.
(121) Keswani MH, Patil AR. The boiled potato peel as a burn wound dressing: a preliminary report. Burns Incl Therm Inj 1985;11(3):220-4.
(122) Subrahmanyam M. Honey dressing versus boiled potato peel in the treatment of burns: a prospective randomized study. Burns 1996;22(6):491-3.
(123) Head Injury. Triage, assessment, investigation and early management of head injury in infants, children and adults. National Collaborating Centre for Acute Care. 2007. Available from: http://www.nice.org.uk/nicemedia/live/11836/36259/36259.pdf.
(124) Kwan I, Bunn F, Roberts I. Spinal immobilisation for trauma patients. Cochrane Database Syst Rev 2001;(2):CD002803.
(125) Hauswald M, Hsu M, Stockoff C. Maximizing comfort and minimizing ischemia: a comparison of four methods of spinal immobilization. Prehosp Emerg Care 2000;4(3):250-2.
(126) Saidi HS, Kahoro P. Experience with road traffic accident victims at The Nairobi Hospital. East Afr Med J 2001;78(8):441-4.
(127) Blackham JE, Claridge T, Benger JR. Can patients apply the Ottawa ankle rules to themselves? Emerg Med J 2008;25(11):750-1.
(128) Omololu AB, Ogunlade SO, Gopaldasani VK. The practice of traditional bonesetting: training algo-rithm. Clin Orthop Relat Res 2008;466(10):2392-8.
(129) Rucinkski TJ, Hooker DN, Prentice WE, Shields EW, Cote-Murray DJ. The effects of intermittent compression on edema in postacute ankle sprains. J Orthop Sports Phys Ther 1991;14(2):65-9.
(130) Brooks SC, Potter BT, Rainey JB. Treatment for partial tears of the lateral ligament of the ankle: a prospective trial. Br Med J (Clin Res Ed) 1981;282(6264):606-7.
(131) Watts BL, Armstrong B. A randomised controlled trial to determine the effectiveness of double Tubigrip in grade 1 and 2 (mild to moderate) ankle sprains. Emerg Med J 2001;18(1):46-50.
(132) Linde F, Hvass I, Jurgensen U, Madsen F. Compression bandage in the treatment of ankle sprains. A comparative prospective study. Scand J Rehabil Med 1984;16(4):177-9.
(133) Thorsson O, Lilja B, Nilsson P, Westlin N. Immediate external compression in the management of an acute muscle injury. Scand J Med Sci Sports 1997;7(3):182-90.
(134) Ernst AA, Thomson T, Haynes M, Weiss SJ. Warmed versus room temperature saline solution for ocular irrigation: A randomized clinical trial. Ann Emerg Med 1998;32(6):676-9.
(135) Corneal superficial injuries. Sowerby Centre for Health Informatics at Newcastle. 2007. Available from: http://www.cks.nhs.uk/corneal_superficial_injury.
(136) Bites - human and animal. Sowerby Centre for Health Informatics at Newcastle. 2007. Available from: http://www.cks.nhs.uk/bites_human_and_animal.

(137) Rittner AV, Fitzpatrick K, Corfield A. Best evidence topic report. Are antibiotics indicated following human bites? Emerg Med J 2005;22(9):654.
(138) Iqbal T, Foex B. Is HIV prophylaxis required in all patients with human bites? BestBETs. 2005. Available from: http://www.bestbets.org/bets/bet.php?id=919.
(139) Teymoortash A, Sesterhenn A, Kress R, Sapundzhiev N, Werner JA. Efficacy of ice packs in the management of epistaxis. Clin Otolaryngol Allied Sci 2003;28(6):545-7.
(140) Scheibe M, Wustenberg EG, Huttenbrink KB, Zahnert T, Hummel T. Studies on the effects of ice collars on nasal blood volume using optical rhinometry. Am J Rhinol 2006;20(4):394-6.
(141) Trotter MI, De R, Drake-Lee A. Evidence-based management of epistaxis in adults. Br J Hosp Med (Lond) 2006;67(12):651-3.
(142) Middleton PM. Epistaxis. Emerg Med Australas 2004;16(5-6):428-40.
(143) Fernandez R, Griffiths R. Water for wound cleansing. Cochrane Database Syst Rev 2008;(1):CD003861.
(144) Zehtabchi S. The role of antibiotic prophylaxis for prevention of infection in patients with simple hand lacerations. Ann Emerg Med 2007;49(5):682-9.
(145) Lacerations. Sowerby Centre for Health Informatics at Newcastle. 2008. Available from: http://www.cks.nhs.uk/lacerations.
(146) Visscher PK, Vetter RS, Camazine S. Removing bee stings. Lancet 1996;348(9023):301-2.
(147) Balit CR, Isbister GK, Buckley NA. Randomized controlled trial of topical aspirin in the treatment of bee and wasp stings. J Toxicol Clin Toxicol 2003;41(6):801-8.
(148) Mackway-Jones K, Teece S. Topical antihistamines for insect bites. BestBETs. 2003. 329 Available from: http://www.bestbets.org/bets/bet.php?id=329.
(149) Vance MV, Selden BS, Clark RF. Optimal patient position for transport and initial management of toxic ingestions. Ann Emerg Med 1992;21(3):243-6.
(150) Poisoning or overdose. Sowerby Centre for Health Informatics at Newcastle. 2007. Available from: http://www.cks.nhs.uk/poisoning_or_overdose.
(151) Intrapartum care: care of healthy women and their babies during childbirth. National Collaborating Centre for Women's and Children's Health. 2007. Available from: http://www.nice.org.uk/nicemedia/pdf/IPCNICEguidance.pdf.
(152) Kamatenesi-Mugisha M, Oryem-Origa H. Medicinal plants used to induce labour during childbirth in western Uganda. J Ethnopharmacol 2007;109(1):1-9.
(153) van Rheenen PF, Brabin BJ. A practical approach to timing cord clamping in resource poor settings. BMJ 2006;333(7575):954-8.
(154) WHO Recommendations for the prevention of postpartum haemorrhage. World Health Organization. 2007. Available from: http://www.who.int/making_pregnancy_safer/publications/WHORecommen-dationsforPPHaemorrhage.pdf.
(155) Edmond KM, Zandoh C, Quigley MA, menga-Etego S, Owusu-Agyei S, Kirkwood BR. Delayed breast feeding initiation increases risk of neonatal mortality. Pediatrics 2006;117(3):e380-e386.
(156) van den Bosch CA, Bullough CH. Effect of early suckling on term neonates' core body temperature. Ann Trop Paediatr 1990;10(4):347-53.
(157) Tawiah-Agyemang C, Kirkwood BR, Edmond K, Bazzano A, Hill Z. Early initiation of breast-feeding in Ghana: barriers and facilitators. J Perinatol 2008;28 Suppl 2:S46-S52.
(158) Bullough CH, Msuku RS, Karonde L. Early suckling and postpartum haemorrhage: controlled trial in deliveries by traditional birth attendants. Lancet 1989;2(8662):522-5.
(159) Irons DW, Sriskandabalan P, Bullough CH. A simple alternative to parenteral oxytocics for the third stage of labor. Int J Gynaecol Obstet 1994;46(1):15-8.
(160) Gebhardt S, Arends E. Standardised maternal guideline on the management of postpartum haemorrhage. S Afr J Obs Gyn 2007;13(3):110-7.

African first aid materials

African
Specialists throughout Sub-Saharan Africa developed the guidelines on which AFAM is based.
All the illustrations are with African models.

Evidence based
The latest scientific first aid guidelines form the fundaments of AFAM.

Efficient
AFAM is ready-to-use and easy-to-use!

Flexible
You can make AFAM yours! Just pick what you need to compose your manual, your first aid courses,... for your target audience. AFAM is a living, user-driven tool.

Professional
Teaching first aid techniques means you take responsibility. Using AFAM means you meet your responsibility since all materials have been validated by experts.

High quality
All situations and techniques are represented by high-resolution quality illustrations. They make AFAM well suited for educational purposes.

R.p.: Philippe Vandekerckhove, Motstraat 40, 2800 Mechelen, Belgium • Version 1.5

"This project breathes life into first aid practice and provides a big push for the development of first aid manuals in Africa" Benson Agbro,
Nigerian Red Cross, member of the AFAM expert panel

"This Red Cross group sets the trend for evidence based guidelines in first aid" Jimmy Volmink,
South African Cochrane Centre, Chair of the AFAM expert panel

Belgian Red Cross
Flanders

Red Cross-Flanders is the Flemish link of the International Red Cross and Red Crescent Movement within the Belgian Red Cross.

Belgian Red Cross-Flanders
Motstraat 40
2800
Mechelen
T +32 (0)15-44 33 22
www.rodekruis.be
